# Synthesis of Nonisocyanate Poly(hydroxy)urethanes from Bis(cyclic carbonates) and Polyamines

**DOI:** 10.3390/polym14132719

**Published:** 2022-07-02

**Authors:** Marc Martínez de Sarasa Buchaca, Felipe de la Cruz-Martínez, Enrique Francés-Poveda, Juan Fernández-Baeza, Luis F. Sánchez-Barba, Andrés Garcés, José A. Castro-Osma, Agustín Lara-Sánchez

**Affiliations:** 1 Departamento de Química Inorgánica, Orgánica y Bioquímica-Centro de Innovación en Química Avanzada (ORFEO-CINQA), Facultad de Ciencias y Tecnologías Químicas, Universidad de Castilla-La Mancha, 13071 Ciudad Real, Spain; marc.martinezsarasa@uclm.es (M.M.d.S.B.); felipe.cruz@uclm.es (F.d.l.C.-M.); enrique.frances@uclm.es (E.F.-P.); juan.fbaeza@uclm.es (J.F.-B.); 2 Departamento de Biología y Geología, Física y Química Inorgánica, Universidad Rey Juan Carlos, 28933 Móstoles, Spain; luisfernando.sanchezbarba@urjc.es (L.F.S.-B.); andres.garces@urjc.es (A.G.); 3 Departamento de Química Inorgánica, Orgánica y Bioquímica-Centro de Innovación en Química Avanzada (ORFEO-CINQA), Facultad de Farmacia, Universidad de Castilla-La Mancha, 02071 Albacete, Spain

**Keywords:** non-isocyanate polyurethanes (NIPUs), poly(hydroxyurethanes) (PHUs), CO_2_, polyaddition reaction, cyclic carbonates

## Abstract

Nonisocyanate polyurethane materials with pending alcohol groups in the polymeric chain were synthesized by polyaddition reaction of bis(cyclic carbonates) onto diamines. For the platform molecule, 1,4-butanediol bis(glycidyl ether carbonate) (BGBC, **1**) was used. The polyaddition reaction of **1** onto a wide range of diamines with different electronic and physical properties was explored. All PHUs were obtained quantitatively after 16 h at 80 °C temperature in MeCN as solvent. The low nucleophilicity of L-lysine has proven unable to ring-open the cyclic carbonate and, thus, no reaction occurred. The addition of DBU or TBD as the catalyst was tested and allows the obtention of the desired PHU. However, the presence of strong bases also led to the formation of polyurea fragments in the new PHU. The different poly(hydroxyurethane) materials were characterized using a wide range of spectroscopic techniques such as NMR, IR, MALDI-ToF, and using GPC studies. The thermal properties of the NIPUs were investigated by DSC and TGA analyses. Moreover, reactions employing different monomer ratios were performed, obtaining novel hydroxycarbamate compounds. Finally, sequential and one-pot experiments were also carried out to synthesize the PHUs polymers in one-step reaction.

## 1. Introduction

**Scheme 1:**
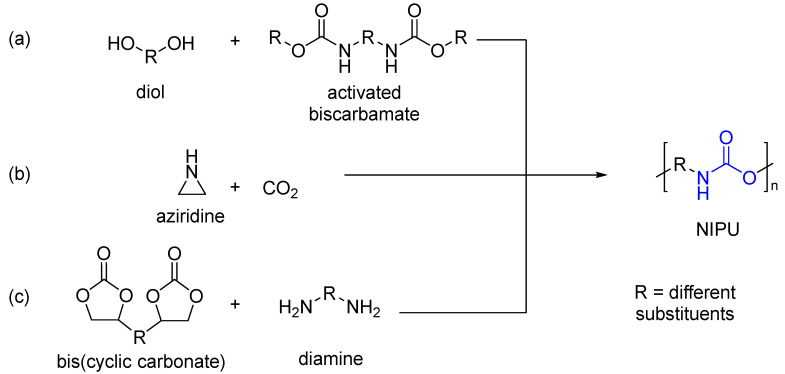
Synthesis of nonisocyanate polyurethanes by: (**a**) transurethanization reaction; (**b**) coupling of azidirines and CO_2_; and (**c**) polyaddition reaction of bis(cyclic carbonates) and diamines.

In the last 50 years, a compelling growth of interest has emerged within the scientific community and plastic industry for the production of polyurethane (PU) materials due to their versatility and wide range of applications. Thanks to their physical properties such as hardness, elongation, strength, abrasion resistance, light weight, etc., they have been widely used in biomedical, building and construction, automotive, textiles, adhesives, packaging, and several other industries [1,2,3,4,5,6,7]. Traditionally, these materials have been prepared by the polyaddition reaction of a diol (or polyol) onto a diisocyanate (or poly isocyanate), and this method is still currently in use in industries [8]. However, isocyanate reagents are hazardous for the environment and their production often requires the use of phosgene, which is highly toxic for humans [9]. Therefore, alternative pathways for PU synthesis involving greener intermediates and processes have become more and more attractive for industrial and academic research. In this context, the synthesis of nonisocyanate polyurethanes can be achieved by the transurethanization polycondensation between a biscarbamate and a diol [6,10,11,12,13], the copolymerization of azidirines and CO_2_ [14,15,16], and the polyaddition reaction of bis(cyclic carbonates) and diamines (Scheme 1) [17,18,19].

**Scheme 2:**
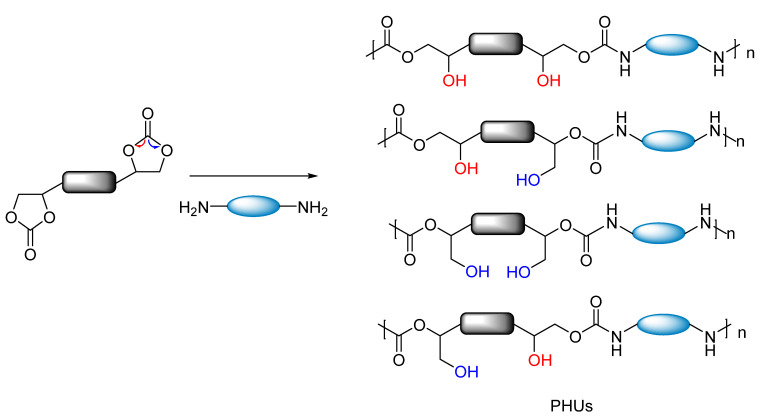
Synthesis of poly(hydroxyurethane)s by polyaddition reaction of bis(cyclic carbonates) and diamines.

Amongst them, most attention has been given to the polyaddition reaction of bis(cyclic carbonates) and diamines since cyclic carbonates have gained much interest from many research groups and industry due to their applications as solvents [20,21], electrolytes for batteries [22,23], and precursors for the synthesis of polymers and fine chemicals [24]. This route exhibits several advantages such as the use of CO_2_ as a sustainable C_1_ feedstock to synthesize the cyclic carbonate, which has been seen of vital environmental importance and as a synthetic challenge during the last few years [25,26,27,28,29,30]. Furthermore, the ring-opening reaction with diamines generates linear poly(hydroxyurethane)s, with pending primary or secondary alcohol groups within the main polymeric chain, which gives the polymer specific properties such as better adhesion, thermal stability, and chemical resistance to nonpolar solvents (Scheme 2) [31]. In addition, the reactive hydroxyl groups enable the polymer to undergo postfunctionalization reactions with chemical and biological functionalities [32]. As reported in the literature, the secondary alcohols moieties are mainly generated during the polymerization process with respect to their primary counterparts [32,33,34].

Since the pioneer work of this synthetic route for the NIPUs in 1957 [35], several reviews about the synthesis of polyurethanes have been reported by different research groups [6,10,17,18,19,31,36]. In general, the ring-opening reaction of the cyclic carbonate by the amine takes place without the use of a catalyst. However, the reaction rate can be accelerated through activation of the monomers by using either weak Lewis acid or oxophilic additives to increase the electrophilicity of the cyclic carbonate group or by the addition of basic additives to increase the nucleophilicity of the amine or even deprotonate it [37,38]. Generally, the polyaddition of cyclic carbonates onto diamines is carried out in polar aprotic solvents such as dimethylformamide (DMF), dimethylsulfoxide (DMSO), N,N-dimethylacetamide (DMAc), etc., due to the better solubility of the starting materials [32,33,39]. The temperature also has an influence on the outcome of the polyaddition reaction [40,41]. Thus, higher yields and reaction rates were obtained when the reaction temperature was increased, which was explained by the decrease of the viscosity in the reaction mixture [41].

In this paper, we reported the synthesis of different NIPUs materials by polyaddition reaction of CO_2_-based cyclic carbonate, 1,4-butanediol bis(glycidyl ether carbonate) 1 (BGBC), with a wide range of commercial diamines to afford the corresponding NIPUs in quantitative yields, including a crosslinked one, which showed enhanced thermal properties. The obtained PHUs were characterized by NMR, IR, MALDI-ToF, and GPC studies. In addition, the synthesis of different carbamates was designed by varying the ratio of the substrates. Finally, one-pot and sequential experiments were also performed to obtain the corresponding PHU materials.

## 2. Experimental Details

### 2.1. Materials and Methods

All manipulations were performed under nitrogen, using standard Schlenk techniques. ^1^H and ^13^C NMR spectra were recorded on a Bruker Ascend TM-500/400 spectrometers (Bruker Corporation, Billerica, MA, USA) and referenced to the residual deuterated solvent. Gel permeation chromatography (GPC) measurements were performed on a Waters 1515 model (Waters Corporation, Milford, CT, USA), equipped with three different columns: 1× PSS GRAM precolumn 10 µm 8 × 50 mm, 1× PSS GRAM column 10 µm 30 Å 8 × 300 mm, and 1× PSS GRAM column 10 µm 1000 Å 8 × 300 mm, and a refractive index detector (Waters 2414). The GPC column was eluted using DMAc as solvent at 25 °C at 1 mL·min^−1^ and calibrated using eight monodisperse polystyrene standards in the range of 580–50000 Da. TGA analysis was performed on a TA instruments TGA-Q50 (TA instruments, New Castle, DE, USA). The heating rate for the sample was 10 °C/min, and the nitrogen flow rate was 60 mL/min. DSC curves were obtained under N_2_ atmosphere on a TA instruments DSC-Q20 (TA instruments, New Castle, DE, USA). Samples were weighed into aluminum crucibles with 5 mg of sample and subjected to two heating cycles at a heating rate of 10 °C/min. The MALDI-ToF spectra were acquired using a Bruker Autoflex II TOF/TOF spectrometer (Bruker Corporation, Billerica, MA, USA) using dithranol (1,8,9-trihydroxyanthracene) as matrix material and NaOAc as additive. Commercially available chemicals were used as received.

### 2.2. Materials and Reagents

Solvents, deuterated solvents, and all other reagents were purchased from common commercial sources and used as received.

### 2.3. General Procedure for the Synthesis of 1,4-Butanediol bis(glycidyl ether carbonate) **1**

The 1,4-butanediol bis-glycidyl ether (BGBE) (50.00 g, 0.25 mol) and the bifunctional organocatalyst (0.99 g, 2.47 mmol) were placed in a 500 mL stainless steel reactor with a magnetic stirrer bar. The reaction mixture was heated to 80 °C, then pressurised to 10 bar of carbon dioxide pressure and stirred for 2 h. The mixture was purified by flash chromatography using a solvent system of first hexane, then hexane:EtOAc (9:1), then hexane:EtOAc (3:1), then EtOAc to achieve the pure cyclic carbonate as a white solid in 90% yield (65.31 g, 0.22 mol).

### 2.4. General Procedure for the Synthesis of Poly(hydroxyurethane)s **1a**-**e**

In a 10 mL Schlenk flask equipped with a small stir bar, BGBC **1** (0.25 g, 0.86 mmol), the corresponding diamine (0.86 mmol), and MeCN (2 mL) were added. The reaction mixture was then warmed up at 80 °C and left stirring for 16 h. After that time, the solvent was removed in vacuo and the residue was washed with methanol. The mixture was filtered, and the solvent was dried in vacuo to afford the corresponding poly(hydroxyurethane) as a rubbery material or a solid depending on the diamine used in 80–95% yield.

### 2.5. Synthesis of Poly(hydroxyurethane) **1f**

The synthesis of PHU **1f** was carried out in a similar manner to PHUs **1a**–**e**, using BGBC (**1**) (0.25 g, 0.86 mmol), *L*-lysine (0.13 g, 0.86 mmol), and 1,5,7-Triazabicyclo[4.4.0]dec-5-ene (TBD) (0.12 g, 0.86 mmol) or 1,8-diazabicyclo[5.4.0]undec-7-ene (DBU) (0.13 g, 0.86 mmol), and **1f** material was obtained as a white-yellowish solid in 93% yield after the appropriate work-up procedure. 

### 2.6. General Procedure for the Synthesis of Hydroxycarbamates **2** and **3**

In a 10 mL Schlenck equipped with a small stir bar, 1,4-butanediol bis(glycidyl ether carbonate) **1** (0.25 g, 0.86 mmol), 1,4-diaminobutane (43 µL, 0.43 mmol and 0.13 mL, 1.72 mmol for **2** and **3** respectively), and MeCN (2 mL) were added. The reaction mixture was then warmed up at 80 °C and left stirring for 16 h. After that time, the solvent was removed in vacuo, extracted with MeOH, and filtered off. Removal of the solvent under vacuum afforded the corresponding hydroxycarbamate as a white solid in 95% yield.

### 2.7. General Procedure for the One-Pot Synthesis of Poly(hydroxyurethane)s **1a,c,d**

The 1,4-butanediol bis-glycidyl ether (BGBE) (0.17 g, 0.86 mmol), bifunctional organocatalyst (3.44 mg, 8.6 μmol) and the corresponding diamine) (0.86 mmol), and MeCN (2 mL) were placed in a 50 mL stainless steel reactor with a magnetic stirrer bar. The reaction mixture was then warmed up at 80 °C, then pressurised to 10 bar of carbon dioxide pressure, and left stirring for 16 h. After that time, CO_2_ was released, the solvent was removed in vacuo and the residue was washed with methanol. The mixture was filtered, and the solvent was dried in vacuo to afford a rubbery mixture composed of the corresponding poly(hydroxyurethane) and polyaminoalcohol was obtained.

### 2.8. General Procedure for the Sequential Synthesis of Poly(hydroxyurethane)s **1a,c,d**

The 1,4-butanediol bis-glycidyl ether (BGBE) (0.17 g, 0.86 mmol) and bifunctional organocatalyst (3.44 mg, 8.6 μmol) were placed in a 50 mL stainless steel reactor with a magnetic stirrer bar. The reaction mixture was then warmed up at 80 °C, then pressurised to 10 bar of carbon dioxide pressure, and left stirring for 2 h. After that time, CO_2_ was released, MeCN (2 mL) and the corresponding diamine (0.86 mmol) were added, and the reaction mixture was stirred for 16 h. Then, the solvent was removed in vacuo and the residue was washed with methanol. The mixture was filtered, and the solvent was dried in vacuo to afford the corresponding poly(hydroxyurethane) **1a**, **1d** and **1e** as rubbery materials in 85–90% yield.

## 3. Results and Discussion

**Scheme 3:**
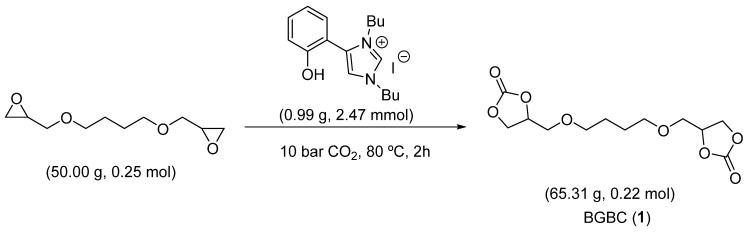
Synthesis of 1,4-butanediol bis(glycidyl ether carbonate) (**1**).

Initially, the multigram scale preparation of 1,4-butanediol bis(glycidyl ether carbonate) (**1**) from the commercially available 1,4-butanediol bis-glycidyl ether and CO_2_ was carried using 1 mol% of the hydroxy-containing imidazole organocatalyst designed by our group [42] at 10 bar of CO_2_ and 80 °C for 2 h under solvent-free conditions affording compound **1** in 90% yield (Scheme 3).

**Scheme 4:**
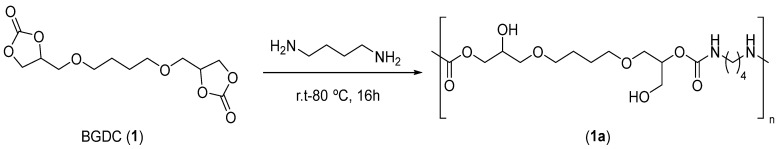
Synthesis of PHU **1a**.

Bis(cyclic carbonate) (**1**) was used along with 1,4-diaminobutane (BDA) in a 1:1 molar ratio to optimize the reaction conditions to synthesize the different NIPUs (Scheme 4). Firstly, the solvent effect on the polyaddition process was investigated (Table 1, entries 1–4). As can be seen, all solvents afforded the formation of the desired PHU quantitatively. However, MeCN was chosen as the optimal solvent to perform this reaction due to its high polarity to increase the solubility of the starting materials and its easy accessibility. Then, the effect of the reaction temperature was studied (Table 1, entries 5–9). As expected, the conversion decreased as the temperature was decreased. Polyaddition of BGBC (**1**) to BDA at room temperature afforded the corresponding PHU in 67% conversion after 16 h of reaction (Table 1, entry 7). This fact has been previously observed for this process. This is probably due to two factors. First, the low reactivity between five members of cyclic carbonates and diamines, and, second, due to monomer diffusion during polymerization. This is an important phenomenon in which are involved the hydrogen bonds created with carbamate groups. The increase of the temperature allows to decrease the viscosity, and to increase the mobility, and thus the advancement of the reaction and the molar masses [17,36,43,44].

**Scheme 5:**
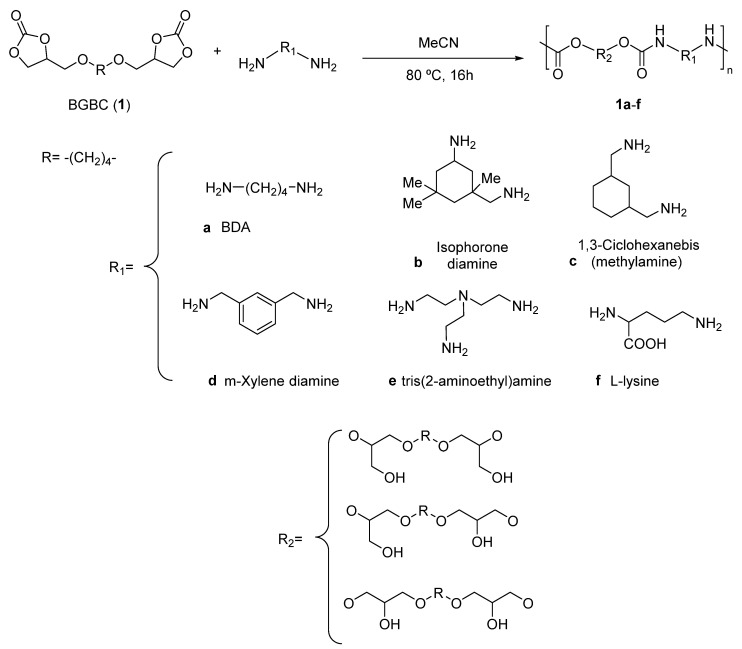
Synthesis of poly(hydroxyurethane)s **1a**-**f**.

**Figure 1:**
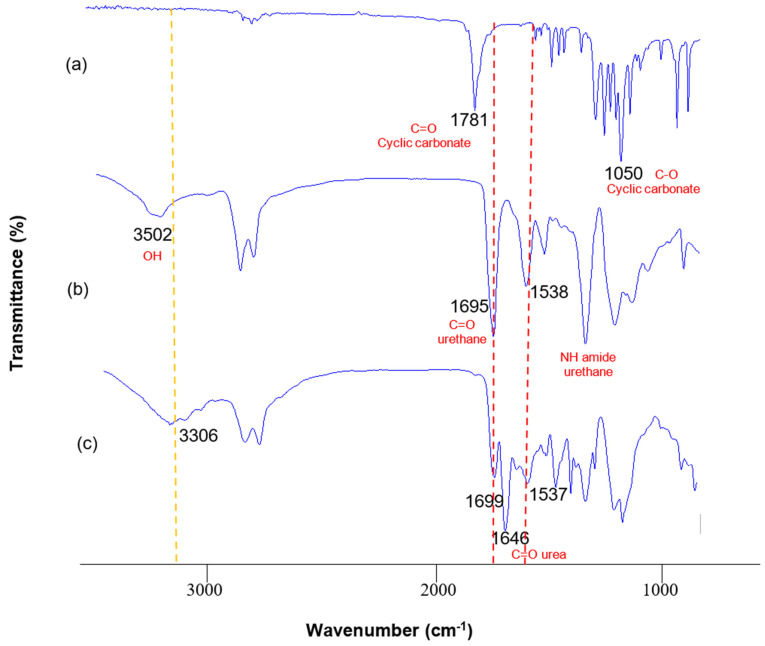
IR spectra for (**a**) BGBC and PHUs (**b**) **1c** and (**c**) **1f**.

Once the optimal conditions for the polyaddition of BGBC (**1**) and BDA were determined (80 °C and MeCN as solvent for 16 h), several PHUs were prepared using a wide range of commercial diamines (Scheme 5). The results are shown in Table 2. All PHUs were obtained quantitatively after 16 h (Table 2, entries 1–6), except when *L*-lysine was used, in which case, no reaction was observed (Table 2, entry 7). This result can be explained based on the low nucleophilicity of *L*-lysine, which is not able to ring-open the cyclic carbonate, and no polyaddition occurred. Since previous works confirmed that the presence of strong bases catalyzed the process [45], the addition of DBU or TBD as catalyst was tested. The addition of a catalyst resulted in the obtention of the desired PHU (Table 2, entries 8 and 9). However, the presence of strong bases also led to the formation of polyurea fragments (Figure 1 and Figure S35), as has been previously reported in similar reactions [38].

The synthesis of a crosslinked PHU was achieved by the reaction of BGBC (**1**) with tris(2-aminoethyl)amine in a 3:2 molar ratio (Table 2, entry 6). The higher *T*_g_ value obtained by differential scanning calorimetry for polymer **1e** suggested the formation of a crosslinked PHU. This high glass transition temperature was attributed to the increased rigidity of the resulting polymer (Table 3, entry 6).

**Figure 2:**
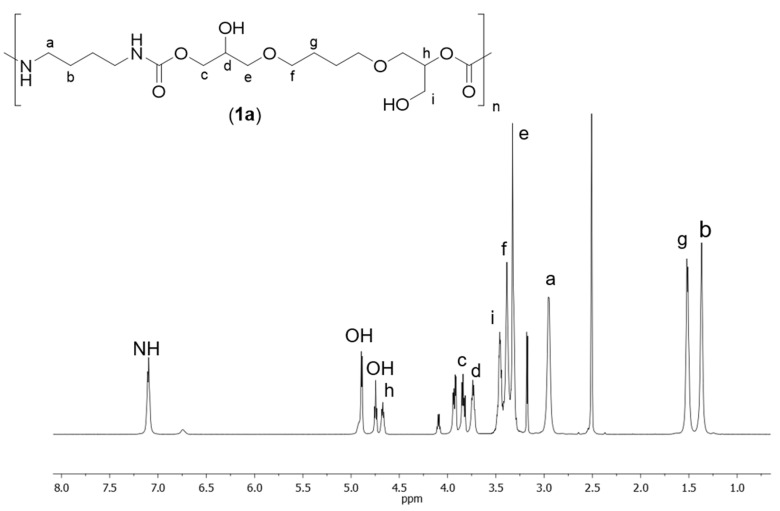
^1^H-NMR spectrum for poly(hydroxyurethane) **1a** in DMSO-d_6_.

**Figure 3:**
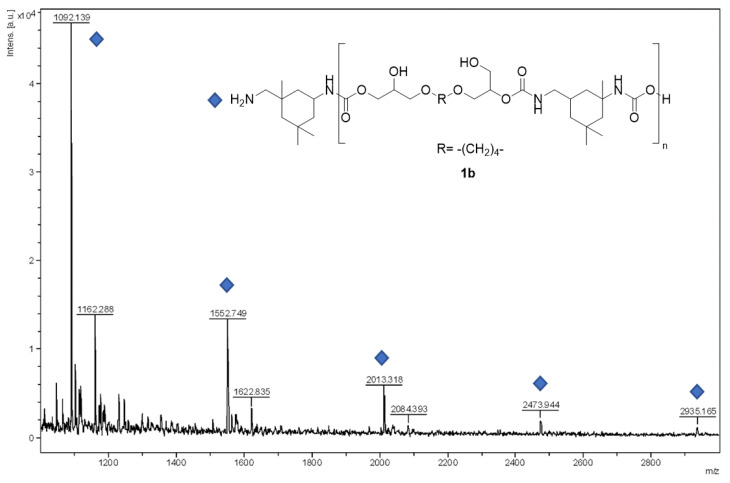
MALDI-TOF spectrum for PHU **1b**.

The chemical structures of the PHUs were characterized by ^1^H-NMR and ^13^C-{^1^H}-NMR (Figure 2 and Supplementary Information), diffusion ordered spectroscopy (DOSY) NMR (Figure S35) and IR spectroscopy (Figure 1 and Supplementary Information), MALDI-ToF analysis (Figure 3 and Supplementary Information), and gel permeation chromatography (GPC) (See Supplementary Information). Figure 1 shows the FT-IR spectra of BGBC (**1**) and PHUs (**1c**) and (**1f**). Bis(glycidyl ether carbonate) (**1**) exhibited two characteristic bands at 1781 and 1050 cm^−1^ corresponding to the cyclic carbonate groups (Figure 1a). However, those bands disappeared in the PHUs IR spectra, and two new bands were observed at 1695 cm^−1^ and 1538 cm^−1^, and 1699 cm^−1^ and 1537 cm^−1^ for PHUs (**1c**) and (**1f**), respectively, confirming the formation of urethane groups (Figure 1b,c, respectively). In addition, another band at 1646 cm^−1^ was observed for PHU (**1f**), which was assigned to the C=O stretching vibration of the urea group, indicating the formation of polyurethane-polyurea chains (Figure 1c) [38]. Analysis of the ^1^H-NMR spectra of the generated polymers also confirmed the formation of the urethane moieties. Characteristic peaks at 7.11 ppm and 6.74 ppm were assigned to the protons of the urethane groups, confirming the urethane structure of the final product (Figure 2). The pair of resonances at 2.96 ppm; 1.37 ppm and 3.39 ppm; 1.51 ppm were assigned to the methylene protons of the alkyl groups of the diamine and the BGBC (**1**), respectively. As has been previously mentioned, it is known that both primary and secondary hydroxyl groups can be formed in the PHU backbone depending on the ring-opening pathway of the cyclic carbonate moiety. In Figure 2, two signals are observed at 4.89 and 4.75 ppm, corresponding to the formation of the secondary and primary hydroxyl groups, respectively. Following previously reported results [32,33,34], the PHUs synthesized in this work exhibited a primary:secondary OH ratio ranging from 28:72 to 38:62 (Table 2, entries 1–5).

MALDI-ToF mass spectrometry was also used to determine the end groups of the obtained PHUs. As an example, the MALDI-ToF spectrum for PHU **1b** (Figure 3) shows a major series of peaks with an *m*/*z* interval of 460 mass units, indicating a controlled alternating microstructure, which is in good agreement with a polymeric chain with one isophorone diamine group in one end and a protonated urethane group in the other end.

Polyhydroxyurethanes **1a**–**e** exhibited molecular weights between 9050 and 17,800 g mol^−1^ with moderate polydispersity ranging from 1.5 to 2.2 (Table 2). This was attributed to the fact that no catalyst was used during the polymerization process. Thus, there is not a good control over the polyaddition reaction and multiple chains with different molecular weights can be generated; hence, the PDI values obtained. On the other hand, molecular weight increased notably for PHU **1f**, since DBU or TBD were added to the polyaddition process, with values of 39,200 g mol^−1^ and 40,325 g mol^−1^, respectively (Table 2, entries 8 and 9).

**Figure 4:**
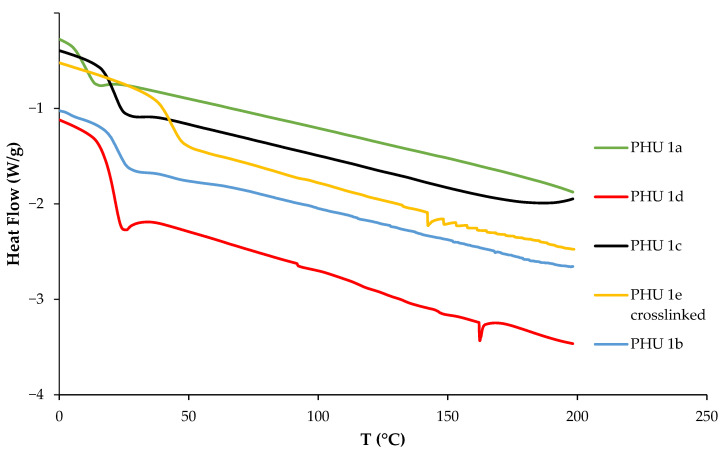
DSC thermograms for selected poly(hydroxyurethanes) PHUs.

Thermal properties of the NIPUs were investigated by DSC and TGA analyses (Table 3). TGA showed that all PHUs were stable in the range of temperatures from 0 °C to 270 °C (Table 3), except for the PHU (**1f**) derived from *L*-lysine (Table 3, entry 7), which was found to be stable between 0 °C and 128 °C. DSC thermograms (Figure 4) revealed that the glass temperature *T*_g_ of the PHUs ranged from −13 °C to 43 °C (Table 3, entries 1–7), depending on the chemical structure of the diamines used. Most studies agreed that higher molecular flexibility between the hydroxyurethane groups led to lower *T*_g_ of PHUs [46,47,48]. Thus, PHUs derived from rigid amines such as isophorone diamine (Table 3, entry 2), 1,3-cyclohexanebis(methylamine) (Table 3, entry 3), or *m*-xylene diamine (Table 3, entry 4) exhibited higher *T*_g_ values than PHUs derived from aliphatic diamines such as BDA (Table 3, entry 1), tris(2-aminoethyl)amine (Table 3, entry 5), or *L*-lysine (Table 3, entry 7). On the other hand, the crosslinked PHU derived from tris(2-aminoethyl)amine **1e** exhibited the highest *T*_g_ (Table 3, entry 6).

**Scheme 6:**
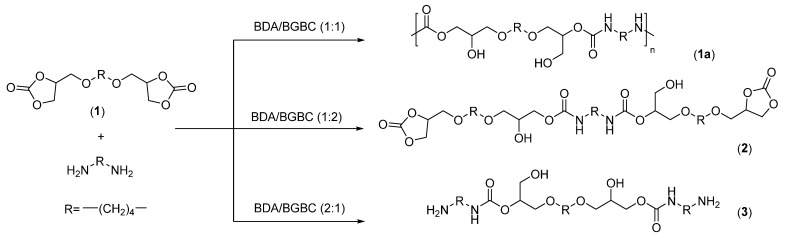
Synthesis of poly(hydroxyurethane)s **1a**–**f**.

The effect of the [diamine]/[BGBC] ratio on the polyaddition reaction of **1** onto 1,4-diaminobutane was also investigated (Scheme 6). Thus, when 0.5 eq. of 1,4-diaminobutane was used (1:2 ratio [diamine]/[BGBC]), a hydroxycarbamate with two cyclic carbonate ending groups was obtained (**2**). On the other hand, when 2 eq. of diamine was added (2:1 ratio [diamine]/[BGBC]), a hydroxycarbamate with two amine ending groups was obtained (**3**). The structures for compounds **2** and **3** were confirmed by NMR and IR spectroscopy and MALDI-ToF analysis. The ^1^H-NMR spectrum for compound **2** exhibited resonances at 4.92 ppm, 4.52 ppm, and 4.27 ppm which confirmed the presence of cyclic carbonate groups, as well as a signal at 7.24 ppm, confirming the formation of the urethane moieties (Figure S38). Additionally, the IR spectrum exhibited two bands at 1787 cm^−1^ and 1048 cm^−1^, which correspond to the cyclic carbonate groups, as well as two bands at 1692 cm^−1^ and 1525 cm^−1^, assigned to the urethane groups generated (Figure S40). The MALDI-ToF spectrum showed single peak with a *m*/*z* of 691 mass units, which is in good agreement with the molecular weight of hydroxycarbamate **2** and Na^+^ (Figure S41).

**Figure 5:**
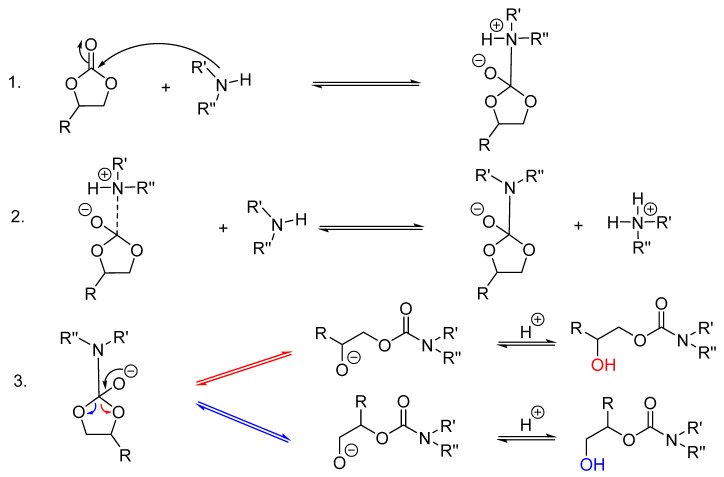
General mechanism proposed for the polymerization process by polyaddition reaction of bis(cyclic carbonates) and diamines.

The results obtained for the reaction of BGBC (**1**) with different diamines support the general mechanism proposed for the polymerization process by polyaddition reaction of bis(cyclic carbonates) and diamines [36,49]. Thus, a three-step reaction involving a tetrahedral intermediate was proposed (Figure 5).

**Scheme 7:**
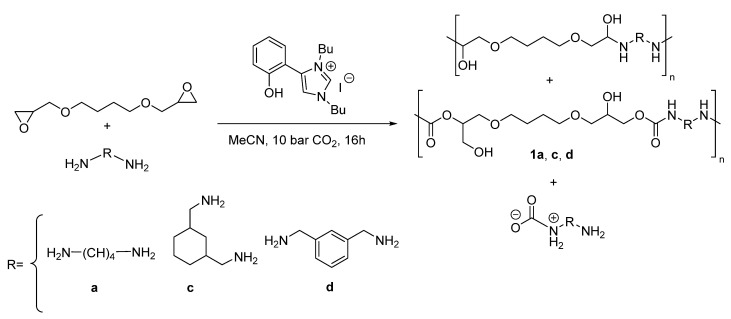
One-pot synthesis of poly(hydroxyurethane)s **1a,c,d**.

After the successful multistep synthesis of PHUs **1a**–**f**, their direct synthesis from BGBE, CO_2_, and a diamine in a one-pot process was investigated (Scheme 7). For that purpose, BDA, 1,3-cyclohexanebis(methylamine), and *m*-xylene diamines were used as starting materials. In all cases, full conversion of BGBE to BGBC (**1**) and subsequent polyaddition onto the corresponding diamine to generate the corresponding NIPU was achieved when the reaction was carried out at 80 °C and 10 bar of CO_2_ for 16 h using acetonitrile as solvent (Scheme 7). Additionally, to polymer **1a**, **1c,** and **1d**, the formation of crosslinked poly(aminoalcohols) from bis-epoxides and diamines was observed, these polymers were previously reported [50,51]. Moreover, a white precipitate was also obtained and was identified as the corresponding carbamate salt from the diamine and CO_2_ reaction (Scheme 7) [52].

**Figure 6:**
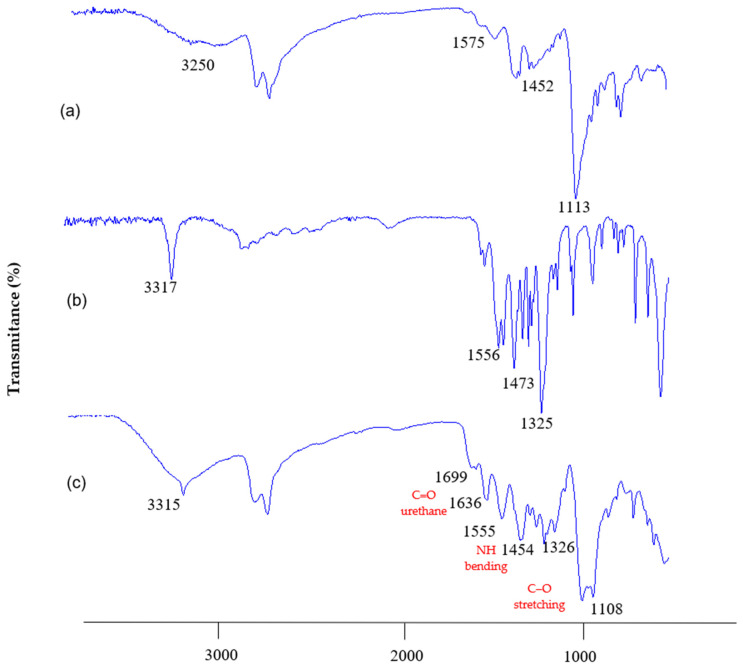
IR spectra for (**a**) 1,4-diaminobutane + BGDE reaction, (**b**) 1,4-diaminobutane + CO_2_ reaction, and (**c**) one pot synthesis of PHU **1a**.

IR analysis of the resulting mixture confirmed the presence of poly(aminoalcohol), carbamate salt and poly(hydroxyurethane). Figure 6 shows the comparison of the IR spectra of a blank sample containing 1,4-diaminobutane and BGBE (Figure 6a), 1,4-diaminobutane and CO_2_ (Figure 6b), and the one-pot reaction mixture (Figure 6c). As can be observed from Figure 5, the one-pot reaction mixture (Figure 6c) shows a sharp band at 3315 cm^−1^ corresponding to the stretching frequency of the amino-end group from the carbamate salt as well as bands at 1555 cm^−1^ and 1326 cm^−1^ which correspond to the N−H bending and C-N stretching frequencies of the amine group. Similarly, the presence of poly(aminoalcohol) was confirmed by the presence of two peaks at 1108 cm^−1^ and 1454 cm^−1^ corresponding to the C-O stretching and O-H bending from the alcohol group. The obtention of the PHU was confirmed by the presence of a band at 1699 cm^−1^ corresponding to the urethane moiety.

**Scheme 8:**
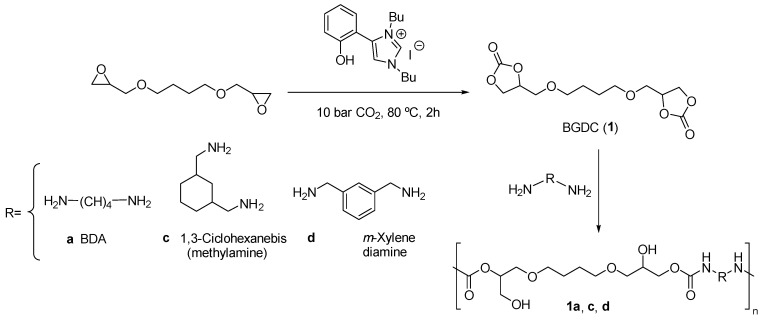
Sequential synthesis of poly(hydroxyurethane)s **1a,c,d**.

Finally, in an effort to minimize the formation of the carbamate salt and the poly(aminoalcohol), a sequential one-pot method was developed for the synthesis of PHUs **1a**,**c**,**d** (Scheme 8). Therefore, bis(cyclic carbonate) BGBC (**1**) was prepared from BGBE as shown in Scheme 3. The subsequent addition of a solution of the diamine in MeCN to the reaction mixture afforded the formation of the corresponding PHUs.

## 4. Conclusions

Herein, we reported the synthesis of different nonisocyanate polyurethanes via the polyaddition reaction of bis(butanediol glycidyl ether)carbonate (**1**) with different diamines under mild reaction conditions. All PHUs were obtained quantitatively after 16 h at 80 °C temperature in MeCN as solvent. The low nucleophilicity of *L*-lysine has proven unable to ring-open the cyclic carbonate and no polyaddition occurred. The addition of DBU or TBD as a catalyst was tested and allows the obtention of the desired PHU. However, the presence of strong bases also led to the formation of polyurea fragments in the new PHU. All polymers were thoroughly characterized by different spectroscopic techniques and their thermal properties were also investigated by DSC and TGA. As expected, crosslinked PHU (**1e**) exhibits the highest glass transition temperature of 43 °C due to its higher rigidity. Under the optimized reaction conditions, molecular weights of the synthesized poly(hydroxyurethane)s showed values up to 18.7 kg mol^−1^ and 40.1 kg mol^−1^ for PHU (**1f**), when an external base was used as catalyst. In addition, the use of different cyclic carbonate/diamines ratios was explored, which allowed the preparation of hydroxycarbamates **2** and **3** quantitatively. One-pot experiments led to the formation of different compounds where CO_2_ carbamate salt and poly(aminoalcohol) products were identified as byproducts. Thus, a sequential one-pot reaction, combining the synthesis of BGBC (**1**) and subsequent polymerization, afforded the formation of the corresponding NIPUs without further purification of the carbonate intermediate.

## Figures and Tables

**Table 1:** Optimization of reaction conditions for the polyaddition of BGBC **1** and 1,4-diaminobutane.

Entry	Solvent	Time (h)	T (°C)	Conversion (%) ^1^
1	DMF	16	80	100
2	MEK	16	80	100
3	EtOAc	16	80	100
4	MeCN	16	80	100
5	MeCN	16	60	93
6	MeCN	16	40	83
7	MeCN	16	r.t	67

^1^ Determined by NMR.

**Table 2:** GPC data for selected PHUs ^1^.

Entry	Diamine (PHU)	Conversion (%) ^2^	Primary OH: Secondary OH	M_n, exp_^3^	PDI ^3^
1	1,4-diaminobutane (**1a**)	100	32:68	14500	2.2
2	Isophorone diamine (**1b**)	100	36:64	13250	1.5
3	1,3-cyclohexanebis (methanamine) (**1c**)	100	36:64	17800	1.6
4	*m*-Xylene diamine (**1d**)	100	38:62	9050	2.2
5	tris(2-aminoethyl)amine (**1e**)	100	28:72	17200	2.1
6 ^4^	tris(2-aminoethyl)amine (**1e**)	100	-	-	-
7	*L*-lysine (**1f**)	0	-	-	-
8 ^5^	*L*-lysine (**1f**)	88	-	39200	2.1
9 ^6^	*L*-lysine (**1f**)	90	-	40325	2.2

^1^ Reactions were carried out at 80 °C in MeCN for 16 h. ^2^ Determined by NMR. ^3^ Determined by GPC. ^4^ 0.66 eq. of tris(2-aminoethyl)amine used. ^5^ 1 eq. of DBU was used. ^6^ 1 eq. of TBD was used.

**Table 3:** DSC and TGA analyses for selected PHUs.

Entry	Diamine (PHU)	*T*_g_ (°C) ^1^	*T*_d, 5%_ (°C) ^2^
1	1,4-diaminobutane (**1a**)	11	252
2	Isophorone diamine (**1b**)	22	272
3	1,3-cyclohexanebis(methylamine) (**1c**)	22	263
4	*m*-Xylene diamine (**1d**)	22	231
5	tris(2-aminoethyl)amine (**1e**)	−3	229
6 ^3^	tris(2-aminoethyl)amine (**1e**)	43	261
7	*L*-lysine (**1f**)	−13	128

^1^ Determined by differential scanning calorimetry. ^2^ Determined by thermogravimetric analysis. ^3^ 0.66 eq. of tris(2-aminoethyl)amine used.

## Supplementary Materials

The following are available online at https://www.mdpi.com/article/10.3390/polym14132719/s1, Figure S1. ^1^H NMR spectrum of PHU **1a** in DMSO-*d*_6_. Figure S2. C^13^{^1^H} NMR spectrum of PHU **1a** in DMSO-*d*_6_. Figure S3. IR spectrum of PHU **1a**. Figure S4. DSC thermogram of PHU **1a**. Figure S5. TGA thermogram of PHU **1a**. Figure S6. ^1^H NMR spectrum of PHU **1b** in DMSO-*d*_6_. Figure S7. C^13^{^1^H} NMR spectrum of PHU **1b** in DMSO-*d*_6_. Figure S8. IR spectrum of PHU **1b**. Figure S9. MALDI-ToF spectrum of PHU **1b**. Figure S10. DSC thermogram of PHU **1b**. Figure S11. TGA thermogram of PHU **1b.** Figure S12. ^1^H NMR spectrum of PHU **1c** in DMSO-*d*_6_. Figure S13. C^13^{^1^H} NMR spectrum of PHU **1c** in DMSO-*d*_6_. Figure S14. IR spectrum of PHU **1c**. Figure S15. DSC thermogram of PHU **1c**. Figure S16. TGA thermogram of PHU **1c**. Figure S17. ^1^H NMR spectrum of PHU **1d** in DMSO-*d*_6_. Figure S18. C^13^{^1^H} NMR spectrum of PHU **1d** in DMSO-*d*_6_. Figure S19. IR spectrum of PHU **1d**. Figure S20. GPC trace of PHU **1d**. Figure S21. DSC thermogram of PHU **1d**. Figure S22. TGA thermogram of PHU **1d**. Figure S23. ^1^H NMR spectrum of PHU **1e** in DMSO-*d*_6_. Figure S24. C^13^{^1^H} NMR spectrum of PHU **1e** in DMSO-*d*_6_. Figure S25. IR spectrum of PHU **1e**. Figure S26. DSC thermogram of PHU **1e**. Figure S27. TGA thermogram of PHU **1e**. Figure S28. IR spectrum of PHU **1e** crosslinked. Figure S29. DSC thermogram of PHU **1e** crosslinked. Figure S30. TGA thermogram of PHU **1e** crosslinked**.** Figure S31. ^1^H NMR spectrum of PHU **1f** in DMSO-*d*_6_. Figure S32. C^13^{^1^H} NMR spectrum of PHU **1f** in DMSO-d_6_. Figure S33. IR spectrum of PHU **1f**. Figure S34. GPC trace of PHU **1f**. Figure S35. DOSY spectrum of PHU **1f**. Figure S36. DSC thermogram of PHU **1f**. Figure S37. TGA thermogram of PHU **1f**. Figure S38. ^1^H NMR spectrum of hydroxycarbamate **2** in DMSO-*d*_6_. Figure S39. C^13^{^1^H} NMR spectrum of hydroxycarbamate **2** in DMSO-*d*_6_. Figure S40. IR spectrum of hydroxycarbamate **2**. Figure S41. MALDI-ToF spectrum of hydroxycarbamate **2**. Figure S42. ^1^H NMR spectrum of hydroxycarbamate **3** in DMSO-d_6_. Figure S43. C^13^{^1^H} NMR spectrum of hydroxycarbamate **3** in DMSO-*d*_6_. Figure S44. IR spectrum of hydroxycarbamate **3**. Figure S45. MALDI-ToF spectrum of hydroxycarbamate **3**.
