# Supplementary material for: Synthesis of Nonisocyanate Poly(hydroxy)urethanes from Bis(cyclic carbonates) and Polyamines
Source: Polymers (Basel). 2022 Jul 2;14(13):2719. doi: 10.3390/polym14132719 (PMC9269535; doi:10.3390/polym14132719)

SUPPORTING INFORMATION FOR:

Synthesis of non-isocyanate poly(hydroxy)urethanes from bis(cyclic carbonates) and polyamines.

Marc Martínez de Sarasa Buchaca[1], Felipe de la Cruz-Martínez[1], Enrique Francés-Poveda[1], Juan Fernández-Baeza[1], Luis F. Sánchez-Barba[3], Andrés Garcés[3], José A. Castro-Osma[2,], and Agustín Lara-Sánchez[1,*]*

[1] Universidad de Castilla-La Mancha, Departamento de Química Inorgánica, Orgánica y Bioquímica-Centro de Innovación en Química Avanzada (ORFEO-CINQA), Facultad de Ciencias y Tecnologías Químicas, 13071-Ciudad Real, Spain. E-mail: Agustin.Lara@uclm.es

[2] Universidad de Castilla-La Mancha, Departamento de Química Inorgánica, Orgánica y Bioquímica-Centro de Innovación en Química Avanzada (ORFEO-CINQA), Facultad de Farmacia, 02071-Albacete, Spain. E-mail: JoseAntonio.Castro@uclm.es

[3] Universidad Rey Juan Carlos, Departamento de Biología y Geología, Física y Química Inorgánica, 28933 Móstoles, Spain.

Correspondence: Agustin.Lara@uclm.es; JoseAntonio.Castro@uclm.es

Table of contents

Structural characterization of PHUs and hydroxycarbamate compounds S3

Figure S1. ^{1}H NMR spectrum of PHU **1a** in DMSO-d_6. S5

Figure S2. $C^{13}\{^{1}H\}$ NMR spectrum of PHU **1a** in DMSO-d_6. S5

Figure S3. IR spectrum of PHU **1a**. S6

Figure S4. DSC thermogram of PHU **1a**. S6

Figure S5. TGA thermogram of PHU **1a**. S7

Figure S6. ^{1}H NMR spectrum of PHU **1b** in DMSO-d_6. S7

Figure S7. $C^{13}\{^{1}H\}$ NMR spectrum of PHU **1b** in DMSO-d_6. S8

Figure S8. IR spectrum of PHU **1b**. S8

Figure S9. MALDI-ToF spectrum of PHU **1b**. S9

Figure S10. DSC thermogram of PHU **1b**. S9

Figure S11. TGA thermogram of PHU **1b**. S10

Figure S12. ^{1}H NMR spectrum of PHU **1c** in DMSO-d_6. S10

Figure S13. $C^{13}\{^{1}H\}$ NMR spectrum of PHU **1c** in DMSO-d_6. S11

Figure S14. IR spectrum of PHU **1c**. S11

Figure S15. DSC thermogram of PHU **1c**. S12

Figure S16. TGA thermogram of PHU **1c**. S12

Figure S17. ^{1}H NMR spectrum of PHU **1d** in DMSO-d_6. S13

Figure S18. $C^{13}\{^{1}H\}$ NMR spectrum of PHU **1d** in DMSO-d_6. S13

Figure S19. IR spectrum of PHU **1d**. S14

Figure S20. GPC trace of PHU **1d**. S14

Figure S21. DSC thermogram of PHU **1d**. S15

Figure S22. TGA thermogram of PHU **1d**. S15

Figure S23. ^{1}H NMR spectrum of PHU **1e** in DMSO-d_6. S16

Figure S24. $C^{13}\{^{1}H\}$ NMR spectrum of PHU **1e** in DMSO-d_6. S16

Figure S25. IR spectrum of PHU **1e**. S17

Figure S26. DSC thermogram of PHU **1e**. S17

Figure S27. TGA thermogram of PHU **1e**. S18

Figure S28. IR spectrum of PHU **1e** crosslinked. S18

Figure S29. DSC thermogram of PHU **1e** crosslinked. S19

Figure S30. TGA thermogram of PHU **1e** crosslinked. S19

Figure S31. ^1H NMR spectrum of PHU **1f** in DMSO-d_6. S20

Figure S32. $C^{13}\{^1H\}$ NMR spectrum of PHU **1f** in DMSO-d_6. S20

Figure S33. IR spectrum of PHU **1f**. S21

Figure S34. GPC trace of PHU **1f**. S21

Figure S35. DOSY spectrum of PHU **1f**. S22

Figure S36. DSC thermogram of PHU **1f**. S22

Figure S37. TGA thermogram of PHU **1f**. S23

Figure S38. ^1H NMR spectrum of hydroxycarbamate **2** in DMSO-d_6. S23

Figure S39. $C^{13}\{^1H\}$ NMR spectrum of hydroxycarbamate **2** in DMSO-d_6. S24

Figure S40. IR spectrum of hydroxycarbamate **2**. S24

Figure S41. MALDI-ToF spectrum of hydroxycarbamate **2**. S25

Figure S42. ^1H NMR spectrum of hydroxycarbamate **3** in DMSO-d_6. S25

Figure S43. $C^{13}\{^1H\}$ NMR spectrum of hydroxycarbamate **3** in DMSO-d_6. S26

Figure S44. IR spectrum of hydroxycarbamate **3**. S26

Figure S45. MALDI-ToF spectrum of hydroxycarbamate **3**. S27

Structural characterization of PHUs and hydroxycarbamates

PHU 1a: ^{1}H-NMR (500 MHz, DMSO-d$_6$): 7.11 (d, J_{HH} = 5.7Hz, 1H, $NH_{urethane}$), 4.89 (d, J_{HH} = 5.1 Hz, 1H, *secondary OH*), 4.75 (t, J_{HH} = 5.5 Hz, 1H, *primary OH*), 4.67 (m, 1H), 3.89 (m, 2H), 3.73 (dd, J_{HH} = 10.4, 5.2 Hz, 1H), 3.46 (m, 2H,), 3.39 (m, 4H, $CH_2{}^{\alpha}{}_{BGDC}$), 3.33 (m, 4H), 2.96 (s, 4H, $CH_2{}^{\alpha}{}_{BDA}$), 1.51 (s, 4H, $CH_2{}^{\beta}{}_{BGDC}$), 1.37 (s, 4H, $CH_2{}^{\beta}{}_{BDA}$). ^{13}C-NMR (500 MHz, DMSO-d$_6$): 156.7 (C=O$_{urethane}$), 73.6, 72.4, 70.9 ($C^{\alpha}{}_{BGDC}$), 69.7, 68.3, 66, 60.6, 40.4 ($C^{\alpha}{}_{BDA}$), 27.2 ($C^{\beta}{}_{BDA}$), 26.4 ($C^{\beta}{}_{BGDC}$). IR: ν (C=O)$_{amide}$ = 1699 cm^{-1}; ν (NH)$_{amide}$ = 1533 cm^{-}1, ν (C–O)$_{urethane}$= 1252 cm^{-1}.

PHU 1b: ^{1}H-NMR (400 MHz, DMSO-d$_6$): 7.11 (s, 1H, NH$_{urethane}$), 4.90 (s, 1H, *OH*), 4.67 (s, 1H), 3.88 (m, 2H), 3.74 (s, 2H), 3.57 (s, 1H, $CH_2{}^{\alpha}$-NH$_{isophorone}$), 3.46 (s, 2H), 3.39 (s, 2H, $CH_2{}^{\alpha}{}_{BGDC}$), 3.32 (s, 2H), 2.72 (d, J$_{H-H}$ = 5.7 Hz, 2H, CH^{β}-NH$_{isophorone}$), 1.52 (s, 2H, $CH_2{}^{\beta}{}_{BGDC}$), 0.93 (m, 11H, CH_3, CH_2 $_{isophorone}$). ^{13}C-NMR (400 MHz, DMSO-d$_6$): 157 (C=O$_{urethane}$), 73.7, 72.4, 70.9 ($C^{\alpha}{}_{BGDC}$), 69.7, 68.3, 66, 60.6, 55 (C^{η}-NH$_{isophorone}$), 47.4 (C$_{isophorone}$), 43.9 (C^{α}-NH$_{isophorone}$), 35.5 (C$_{isophorone}$), 28.4 (C$_{isophorone}$), 26.4($C^{\beta}{}_{BGDC}$), 23.7 (C$_{isophorone}$). IR: ν (C=O)$_{amide}$ = 1695 cm^{-1}; ν (NH)$_{amide}$ = 1537 cm^{-1}, ν (C–O)$_{urethane}$ = 1243 cm^{-1}.

PHU 1c: ^{1}H-NMR (500 MHz, DMSO-d$_6$): 7.11 (s, 1H, NH$_{urethane}$), 4.89 (s, 1H, *secondary OH*), 4.75 (s, 1H, *primary OH*), 4.67 (m, 1H), 3.88 (ddd, , J_{HH} = 15.2, 10.9, 6 Hz), 3.73 (m, 2H), 3.46 (s, 2H), 3.39 (s, 2H, $CH_2{}^{\alpha}{}_{BGDC}$), 3.32 (s, 2H), 2.95 (d, J_{HH} = 5.1 Hz, 1H), 2.80 (d, J_{HH} = 3.6 Hz, 4H, CH^{σ}-NH$_{diamine}$), 1.67 (d, J_{HH} = 12.9 Hz, 2H), 1.51 (d, J_{HH} = 5.9 Hz, 4H, $CH_2{}^{\beta}{}_{BGDC}$), 1.37 (s, 2H, $CH_2{}^{\beta}$-NH$_{diamine}$), 1.27 (dd, J_{HH} = 13, 8.1Hz, 1H), 1.13 (m, 1H), 0.73 (m, 1H), 0.47 (q, J_{HH} = 12.2 Hz, 1H). ^{13}C-NMR (500 MHz, DMSO-d$_6$): 156.9 (C=O$_{urethane}$), 73.5, 72.4, 70.9 ($C^{\alpha}{}_{BGDC}$), 69.7, 68.3, 66, 60.6, 47.3 (C^{α}-NH$_{diamine}$), 38 (C^{β}-NH$_{diamine}$), 30.8 (C$_{diamine}$), 26.4 ($C^{\beta}{}_{BGDC}$). IR: ν (C=O)$_{amide}$ = 1695 cm^{-1}; ν (NH)$_{amide}$ = 1538 cm^{-1}, ν (C–O)$_{urethane}$ = 1251 cm^{-1}.

PHU 1d: ^{1}H-NMR (400 MHz, DMSO-d$_6$): 7.67 (s, 1H, NH$_{urethane}$), 7.26 (t, J_{HH} = 7.7Hz, 1H, $Ph_{m\text{-}xylene}$), 7.13 (d, J_{HH} = 7.2 Hz, 3H, $Ph_{\alpha\text{-}xylene}$), 4.92 (s, 1H, *secondary OH*), 4.78 (s, 1H, *primary OH*), 4.72 (m, 1H), 4.16 (d, J_{HH} = 5.8 Hz, 2H, $CH_2{}^{\alpha}$-NH$_{m\text{-xylene}}$), 3.93 (ddd, , J_{HH} = 17.2, 11, 5.3 Hz, 2H), 3.76 (s, 1H), 3.48 (s, 2H), 3.39 (s, 2H, $CH_2{}^{\alpha}{}_{BGDC}$), 3.34 (s, 2H), 1.52 (s, 4H, $CH_2{}^{\beta}{}_{BGDC}$). ^{13}C-NMR (500 MHz, DMSO-d$_6$): 157 (C=O$_{urethane}$), 140.3 ($Ph_{m\text{-}xylene}$), 128.7 ($Ph_{m\text{-}xylene}$), 126.7 ($Ph_{m\text{-}xylene}$), 126 ($Ph_{m\text{-}xylene}$), 73.9, 72.4, 70.9 ($C^{\alpha}{}_{BGDC}$), 69.7, 68.3, 66.2, 60.6, 44.3 (C^{α}-NH$_{m\text{-xylene}}$), 26.4 ($C^{\beta}{}_{BGDC}$). IR: ν (C=O)$_{amide}$ = 1695 cm^{-1}; ν (NH)$_{amide}$ = 1533 cm^{-1}, ν (C–O)$_{urethane}$ = 1247 cm^{-1}.

PHU 1e: ^{1}H-NMR (500 MHz, DMSO-d$_6$): 7.15 (s, 1H, NH$_{urethane}$), 4.68 (dt, J$_{H-H}$ = 10; 4.9 Hz, 1H), 3.90 (ddd, , J_{HH} = 17.8, 10, 7 Hz, 2H), 3.75 (m, 1H), 3.46 (s, 2H), 3.39 (s, 2H, $CH_2{}^{\alpha}{}_{BGDC}$), 3.32 (d, J_{HH} = 5.4Hz, 2H), 3.01 (d, J_{HH} = 5.6Hz, 4H, $CH_2{}^{\alpha}$-NH$_{urethane}$), 2.55 (m, 2H, $CH_2{}^{\alpha}$-NH$_{triamine}$), 2.45 (m, 4H, $CH_2{}^{\beta}$-NH$_{urethane}$), 2.38 (dd, J_{HH} = 13.1, 6.7 Hz, 2H, $CH_2{}^{\beta}$-NH$_{triamine}$), 1.52 (m, 2H, $CH_2{}^{\beta}{}_{BGDC}$). ^{13}C-NMR (500 MHz, DMSO-d$_6$): 156.9 (C=O$_{urethane}$), 73.9, 72.4, 70.9 ($C^{\alpha}{}_{BGDC}$), 69.7, 68.2, 66.2, 60.6, 58.4

(C^{β}-NH$_{urethane}$), 54.4 (C^{β}-NH$_{triamine}$), 40 (C^{α}-NH$_{triamine}$), 39.5(CH_2^{α}-NH$_{urethane}$), 26.4 ($C^{\beta}$$_{BGDC}$). IR: ν (C=O)$_{amide}$ = 1696 cm^{-1}; ν (NH)$_{amide}$ = 1532 cm^{-1}, ν (C–O)$_{urethane}$ = 1251 cm^{-1}.

PHU 1f: ^{1}H-NMR (500 MHz, DMSO-d$_6$): 7.13 (s, 1H, $NH_{urethane}$), 6.21 (s, 1H, NH_{urea}), 4.65 (s, 1H), 3.86 (m, 2H), 3.82 (m, 1H, CH^{α}-NH$_{L\text{-}lysine}$), 3.75 (m, 1H), 3.46 (s, 2H), 3.39 (s, 2H, $CH_2^{\alpha}$$_{BGDC}$), 3.32 (d, J_{HH} = 5.4Hz, 2H), 3.26-3.3.20 (m, 8H, *DBU*), 2.91 (d, J_{HH} = 5.4 Hz, 4H, CH_2^{α}-NH$_{L\text{-}lysine}$), 2.61 (d, J_{HH} = 5.4 Hz, 2H, *DBU*), 1.85 (m, 2H, *DBU*), 1.64-1.60 (m, 4H, *DBU*), 1.51 (s, 2H, $CH_2^{\beta}$$_{BGDC}$), 1.32 (m, 4H, CH_2^{β}-NH$_{L\text{-}lysine}$), 1.19 (s, 2H, CH_2^{g}-NH$_{L\text{-}lysine}$). ^{13}C-NMR (500 MHz, DMSO-d$_6$): 174.1(C=$O_{L\text{-lysine}}$), 164.7 (*DBU*), 156.7 (C=$O_{urethane}$), 155.8 (C=O_{urea}), 73.9, 72.4, 70.9 ($C^{\alpha}$$_{BGDC}$), 69.7, 68.2, 66.0, 60.6, 56.0 (C^{α}-NH$_{L\text{-}lysine}$), 53.4 (*DBU*), 48.22 (*DBU*), 40.9 (C^{α}-NH$_{L\text{-}lysine}$), 32.9 (C^{β}-NH$_{L\text{-}lysine}$), 32.7 (*DBU*), 30.0 (C^{β}-NH$_{L\text{-}lysine}$), 27.0(*DBU*), 26.4 ($C^{\eta}$$_{BGDC}$), 24.5 (*DBU*), 22.8 ($C^{\gamma}$-NH$_{L\text{-}lysine}$), 20.2 (*DBU*). IR: ν (C=O)$_{amide}$ = 1697 cm^{-1} ; ν (C=O)$_{urea}$ = 1646 cm^{-1}, ν (NH)$_{amide}$ = 1537 cm^{-1}, ν (C–O)$_{urethane}$ = 1245 cm^{-1}.

Hydroxycarbamate 2: ^{1}H-NMR (400 MHz, DMSO-d$_6$): 7.11 (d, $J_{H\text{-}H}$ = 5.5Hz, 1H, $NH_{urethane}$), 4.94 (m, 2H, CH $_{cyclic\ carbonate}$), 4.75 (t, $J_{H\text{-}H}$ = 5.5 Hz, 1H, *primary OH*), 4.67 (m, 1H), 4.52 (t, J_{HH} = 8.4Hz, 2H, $CH_{2,cyclic\ carbonate}$), 4.25 (dd, J_{HH} = 8.2, 5.9Hz, 2H, $CH_{2,cyclic\ carbonate}$), 4.09 (q, J_{HH} = 5.2 Hz, 1H, *secondary OH*), 3.89 (dd, J_{HH} = 27.1, 5.3Hz, 2H), 3.74 (m, 1H), 3.59 (ddd, J_{HH} = 15.6, 11.5, 3.4Hz, 4H), 3.46 (m, 2H), 3.39 (m, 4H, $CH_2^{\sigma}$$_{BGDC}$), 3.33 (m, 2H), 2.95 (s, 4H, $CH_2^{\alpha}$$_{BDA}$), 1.53 (s, 4H, $CH_2^{\beta}$$_{BGDC}$), 1.37 (s, 4H, $CH_2^{\eta}$$_{BDA}$). ^{13}C-NMR (400 MHz, DMSO-d$_6$): 156.7 (C=$O_{urethane}$), 155.4 (C=$O_{cyclic\ carbonate}$), 76.0 ($CH_{cyclic\ carbonate}$), 73.6, 72.4, 71.0, 70.9 ($C^{\sigma}$$_{BGDC}$), 70.0, 68.3, 66.6 ($CH_{2,\ cyclic\ carbonate}$), 66.0, 60.6, 40.4 ($C^{\alpha}$$_{BDA}$), 27.2 ($CH_2^{\beta}$$_{BDA}$), 26.1 ($CH_2^{\beta}$$_{BGDC}$). IR: ν (C=O)$_{cyclic\ carbonate}$ = 1787 cm^{-1}, ν (C=O)$_{amide}$ = 1692 cm^{-1}; ν (NH)$_{amide}$ = 1525 cm^{-1}, ν (C–O)$_{urethane}$ = 1249, 1049 cm^{-1}.

Analysis found:	C: 50.5 %	H: 7.5 %	N: 3.9 %
Calculated:	C: 50.3 %	H: 7.2 %	N: 4.2 %

Hydroxycarbamate 3: ^{1}H-NMR (400 MHz, DMSO-d$_6$): 7.11 (s, 1H, $NH_{urethane}$), 4.68 (m, 1H), 3.88 (ddd, , J_{HH}= 17.1, 11, 5.4Hz, 4H, *2*), 3.73 (m, 1H), 3.46 (m, 2H, *11*), 3.39 (m, 4H, $CH_2^{\alpha}$$_{BGDC}$), 3.32 (m, 4H), 2.95 (dd, J_{HH}= 12.6, 6.5Hz, 4H, $CH_2^{\alpha}$$_{BDA}$), 2.5 (m, 4H, $CH_2^{\alpha}$$_{BDA,urethane}$), 1.51 (d, J_{HH}= 4.6Hz, 4H, $CH_2^{\beta}$$_{BGDC}$), 1.35 (m, 4H, $CH_2^{\beta}$$_{BDA,BDA\ urethane}$ *3*). ^{13}C-NMR (400 MHz, DMSO-d$_6$): 156.7 (C=O$_{urethane}$), 73.5, 72.4, 70.9 ($C^{\alpha}$$_{BGDC}$), 69.7, 68.3, 66.0, 60.6, 41.9 ($C^{\alpha}$$_{BDA,\ urethane}$), 40.4 ($C^{\alpha}$$_{BDA}$), 31.1 ($C^{\beta}$$_{BDA,\ urethane}$) 27.5 ($C^{\beta}$$_{BDA}$), 26.4 ($C^{\beta}$$_{BGDC}$). IR: ν (O–H) = 3340 cm^{-1}, ν (C=O)$_{amide}$ = 1687 cm^{-1}; ν (NH)$_{amide}$ = 1535 cm^{-}1, ν (C–O)$_{urethane}$ = 1248, 1109 cm^{-1}.

Analysis found:	C: 52.7 %	H: 9.5 %	N: 11.4 %
Calculated:	C: 52.5 %	H: 9.2 %	N: 11.7 %

Figure S1. ^{1}H-NMR spectrum of PHU **1a** in DMSO-d$_6$.

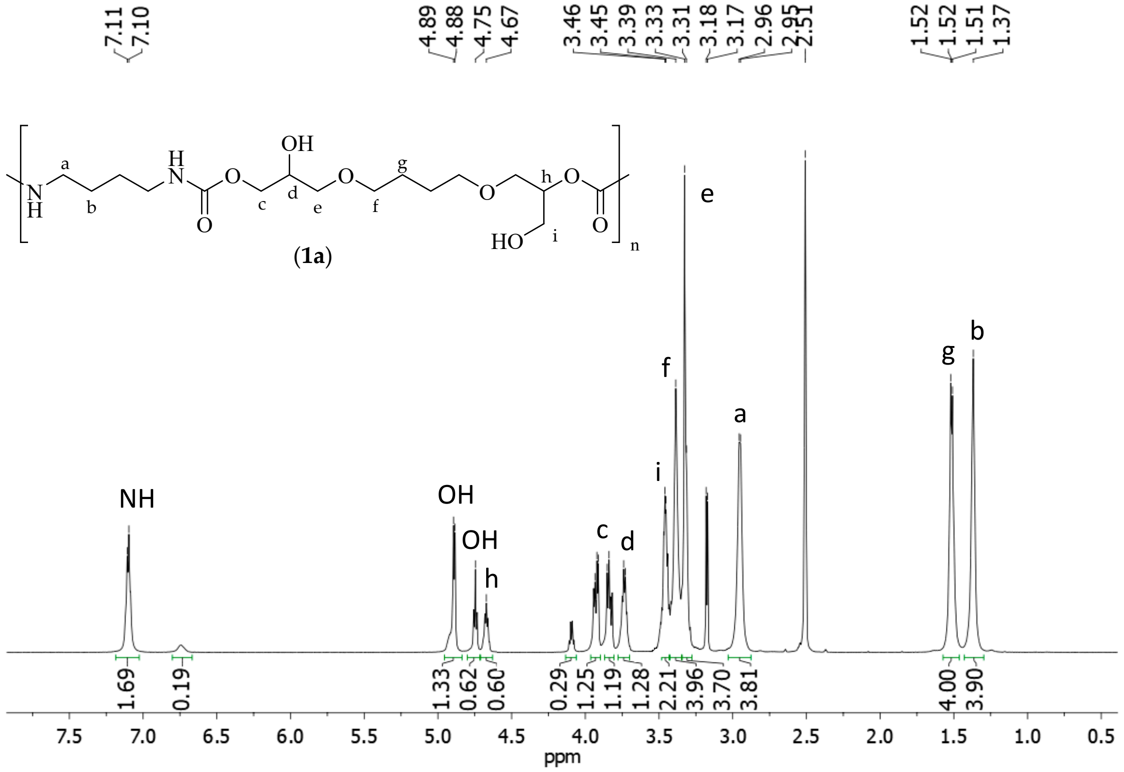

Figure S2. $^{13}C-\{^{1}H\}$-NMR spectrum of PHU **1a** in DMSO-d$_6$.

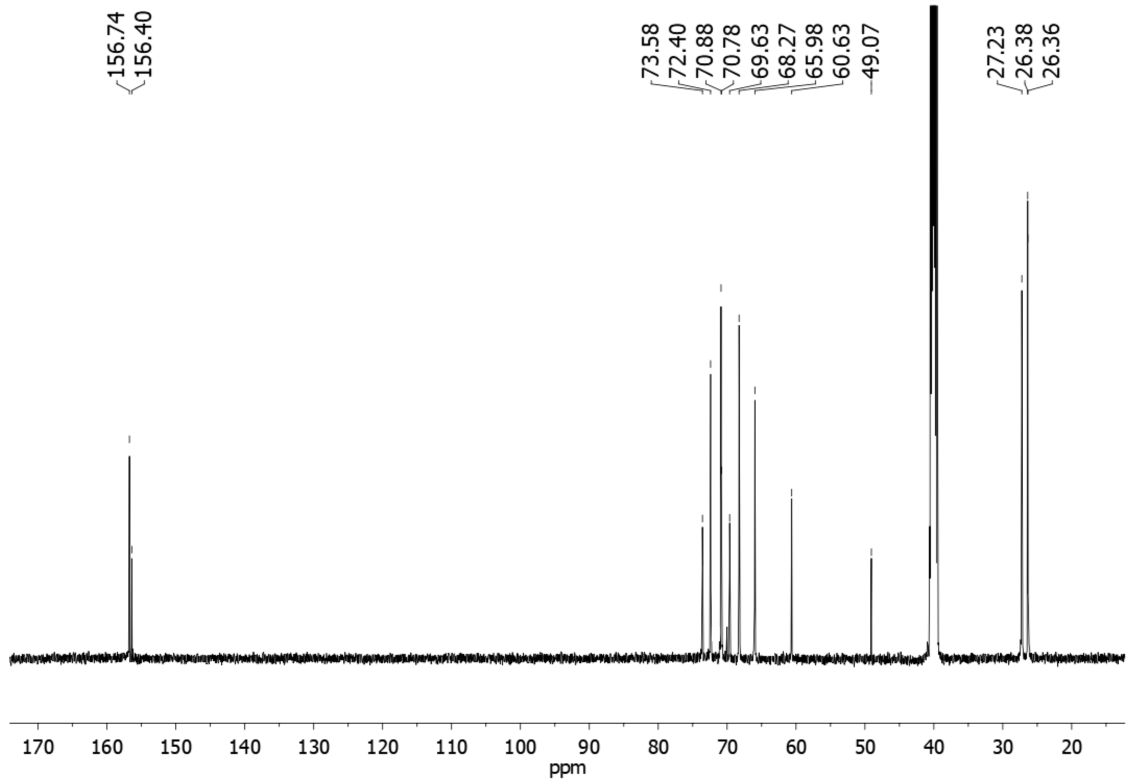

Figure S3. IR spectrum of PHU **1a**.

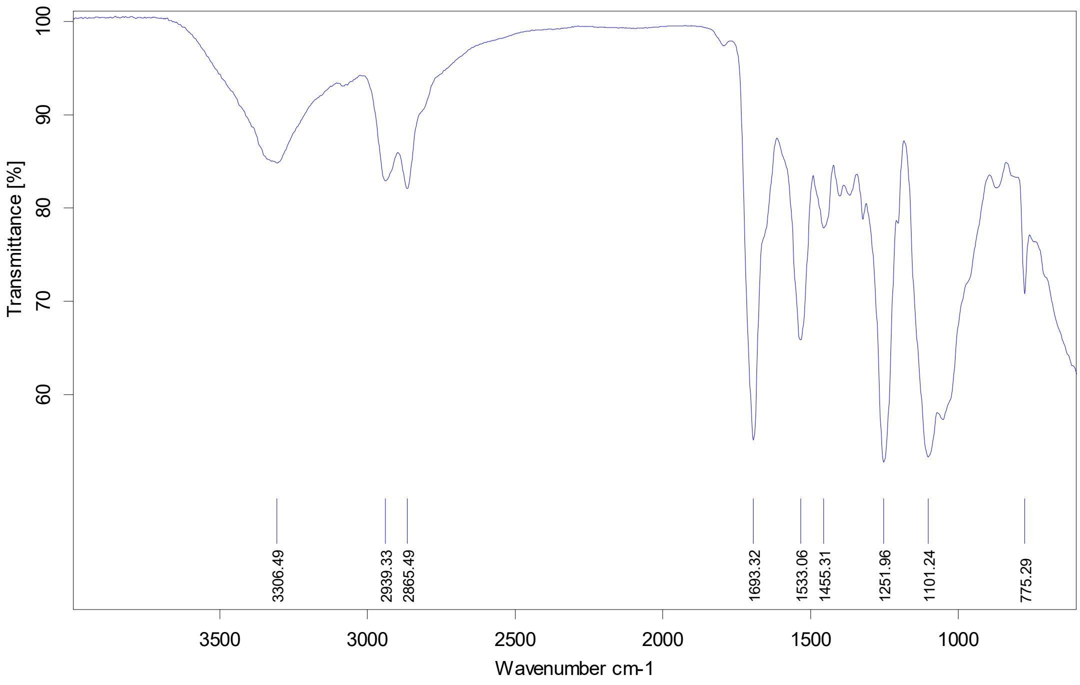

Figure S4. DSC thermogram of PHU **1a**.

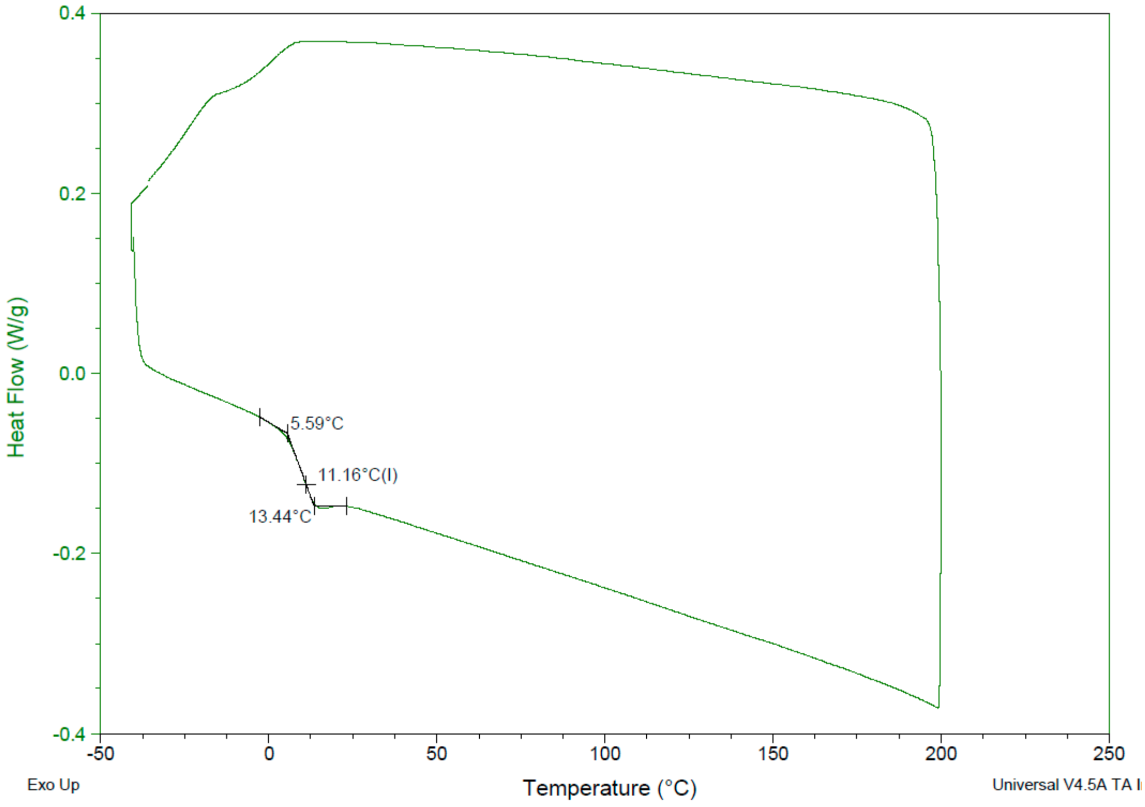

Figure S5. TGA thermogram of PHU **1a**.

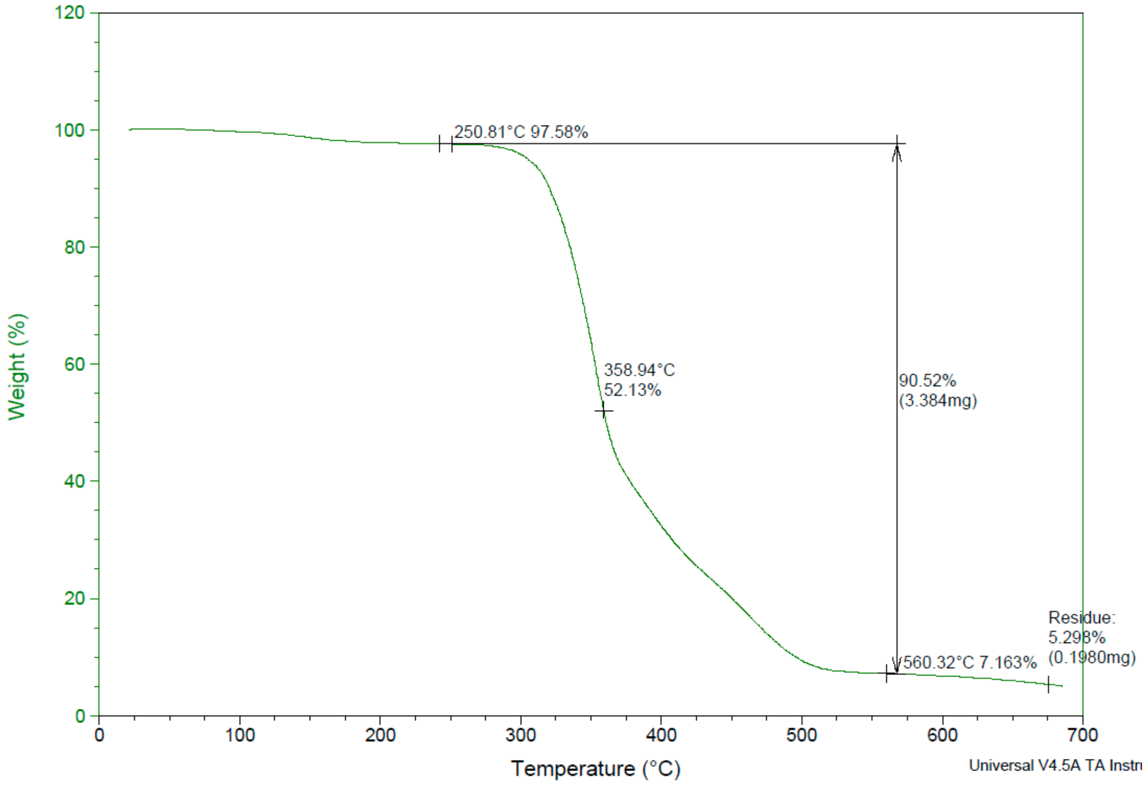

Figure S6. ^{1}H-NMR spectrum of PHU **1b** in DMSO-d6.

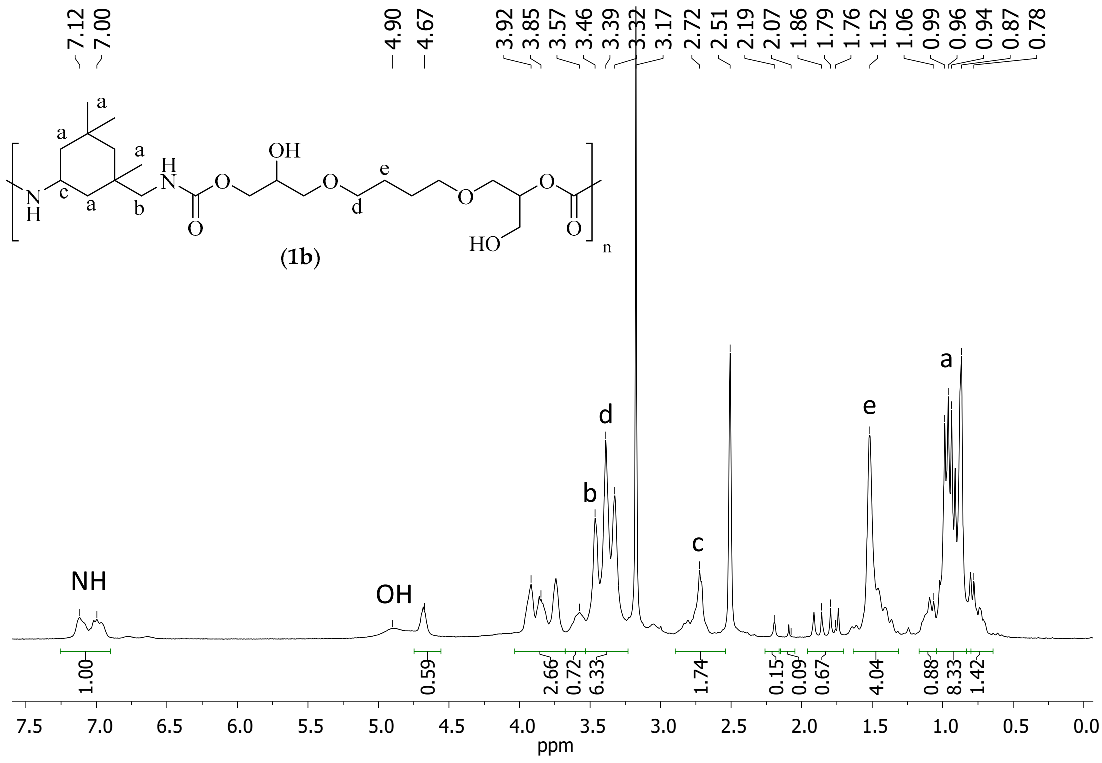

Figure S7. ^{13}C-{^{1}H}-NMR spectrum of PHU **1b** in DMSO-d$_6$.

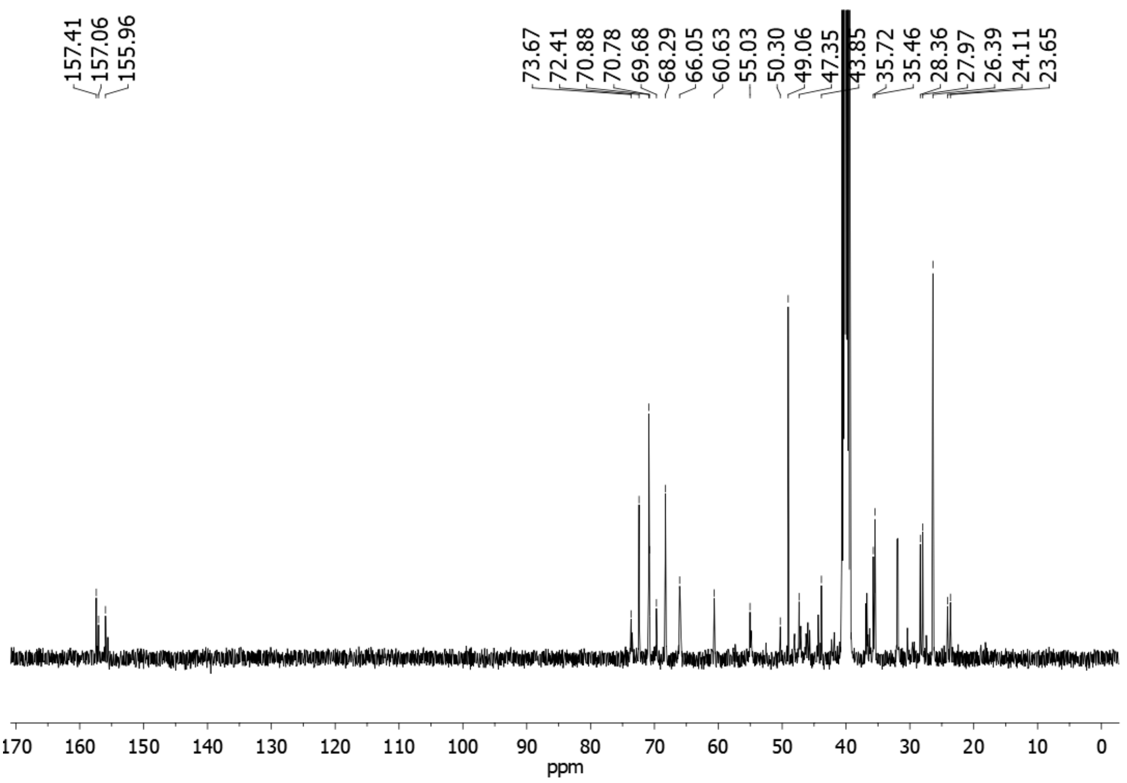

Figure S8. IR spectrum of PHU **1b**.

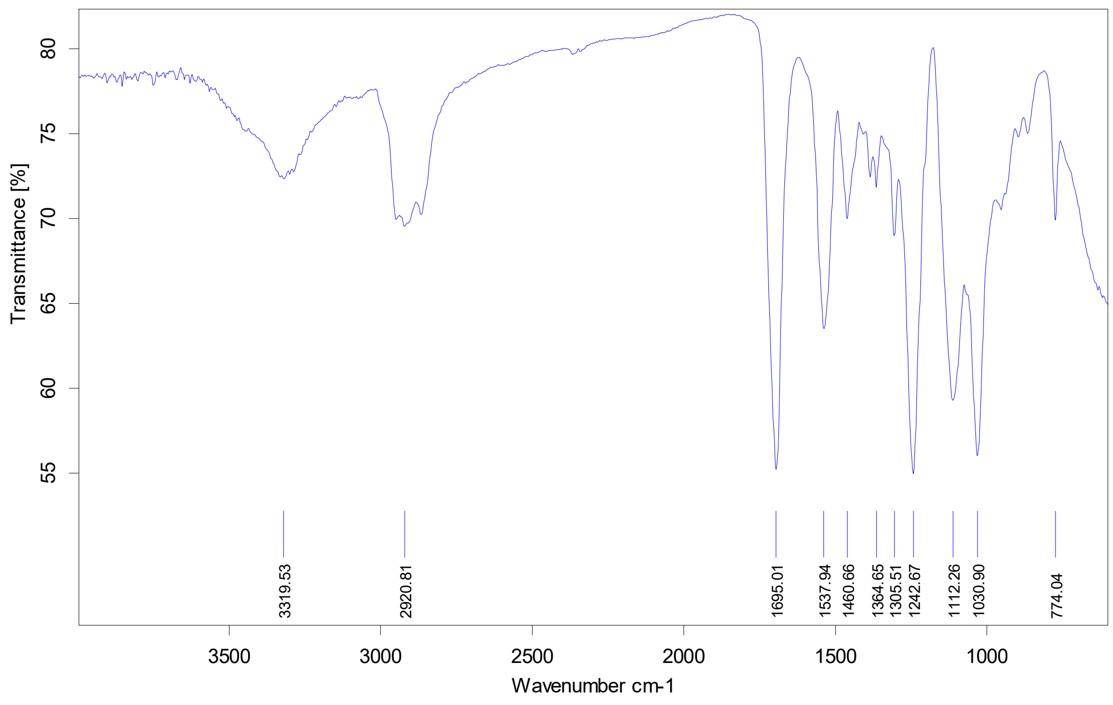

Figure S9. MALDI-ToF spectrum of PHU **1b**.

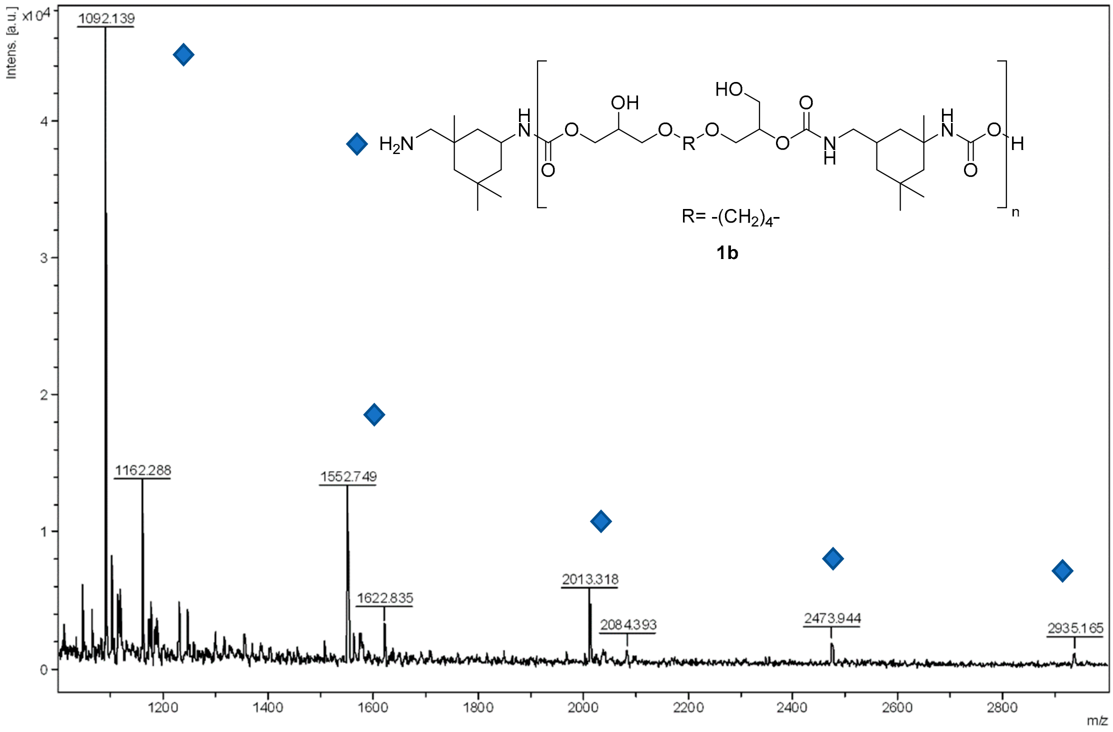

Figure S10. DSC thermogram of PHU **1b**.

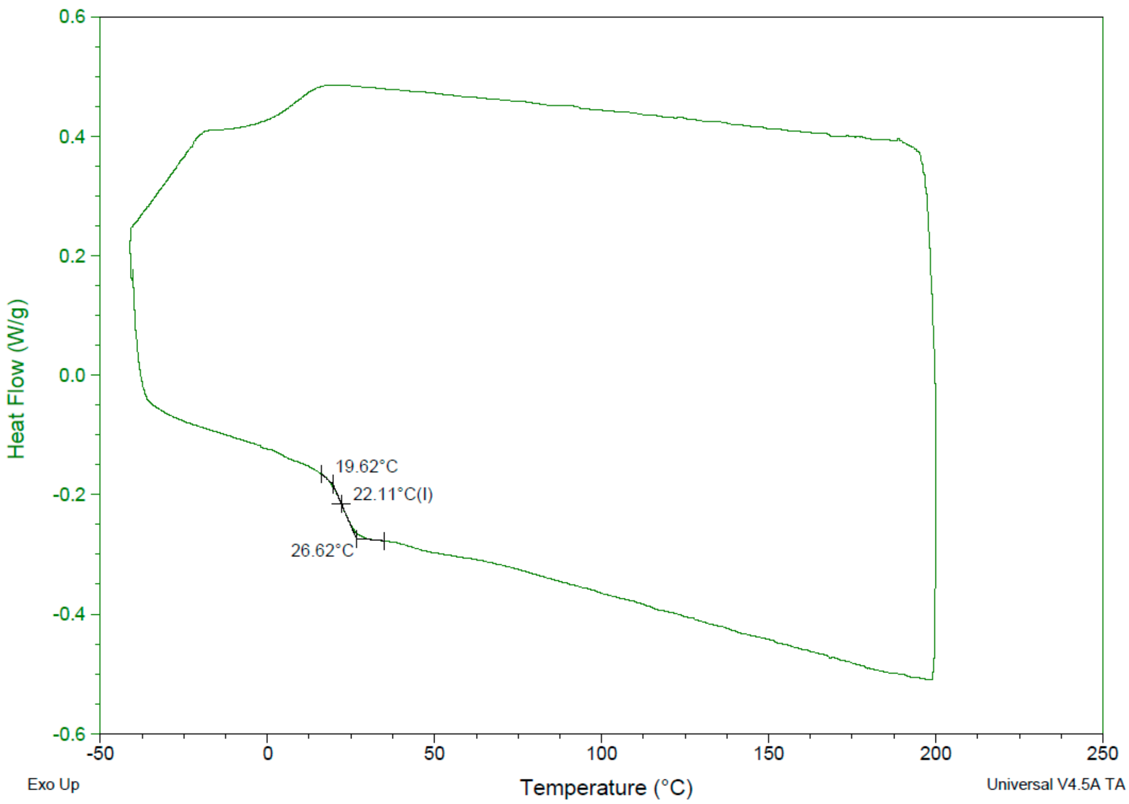

Figure S11. TGA thermogram of PHU **1b**.

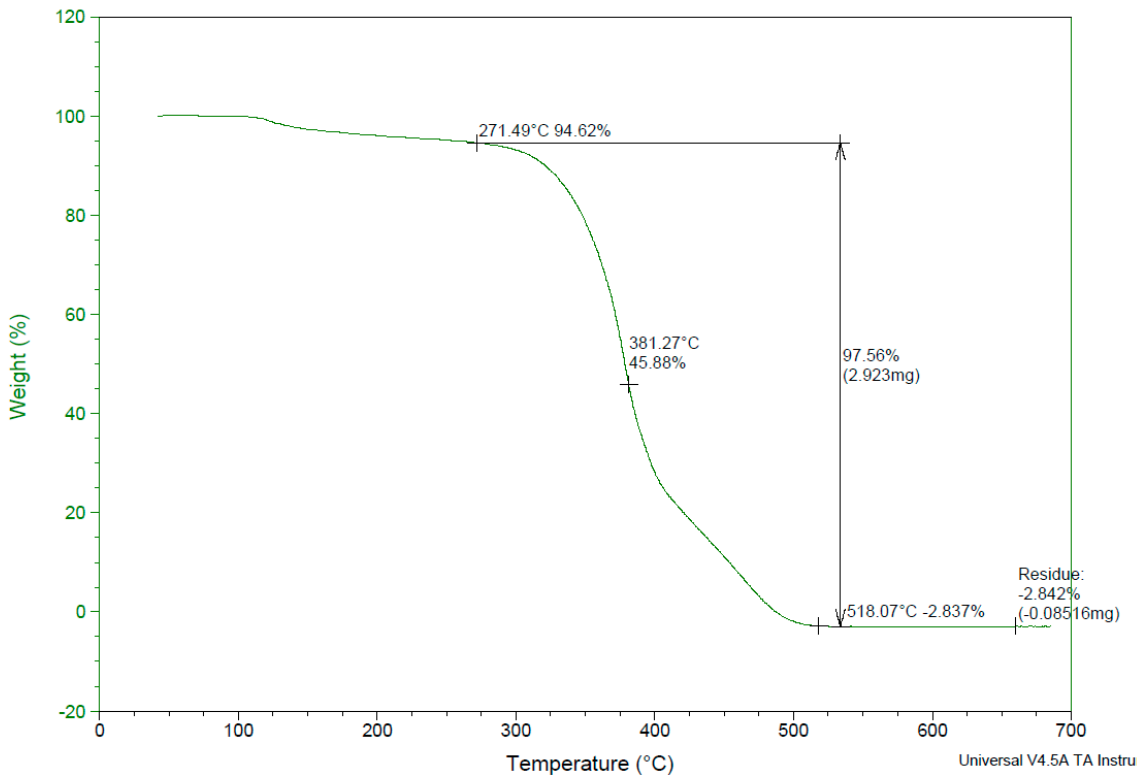

Figure S12. ^{1}H-NMR spectrum of PHU **1c** in DMSO-d6.

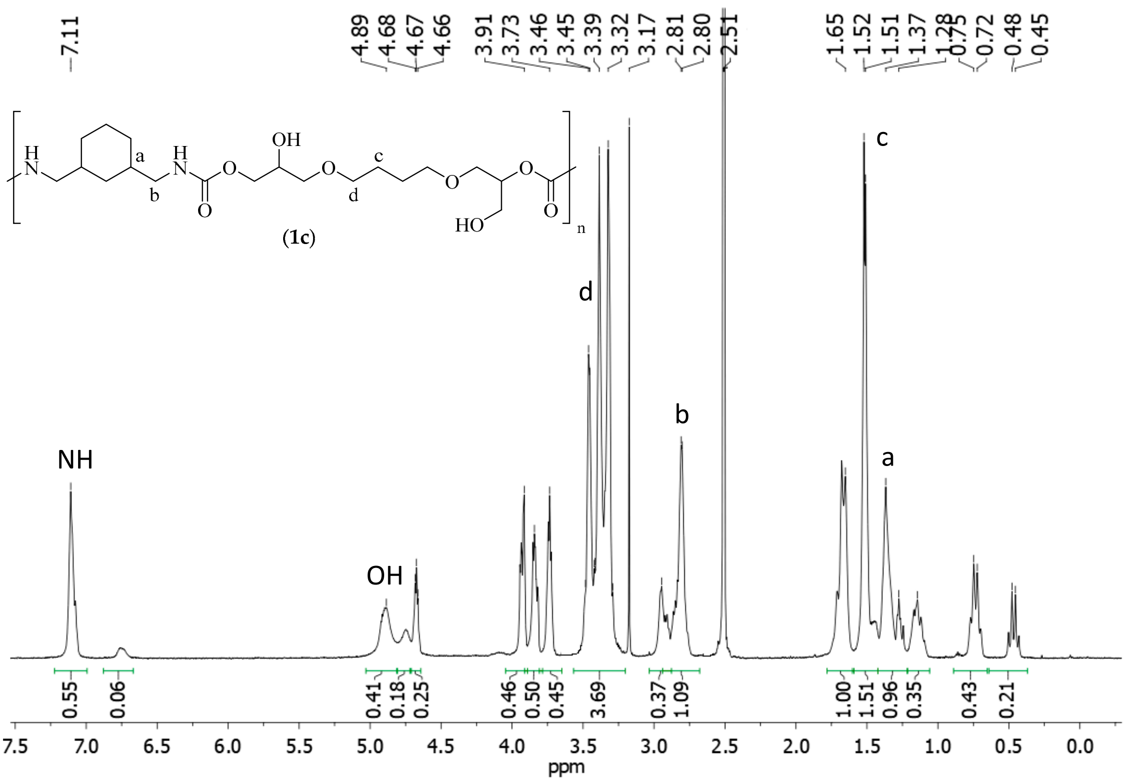

Figure S13. ^{13}C-{^1H}-NMR spectrum of PHU **1c** in DMSO-d$_6$.

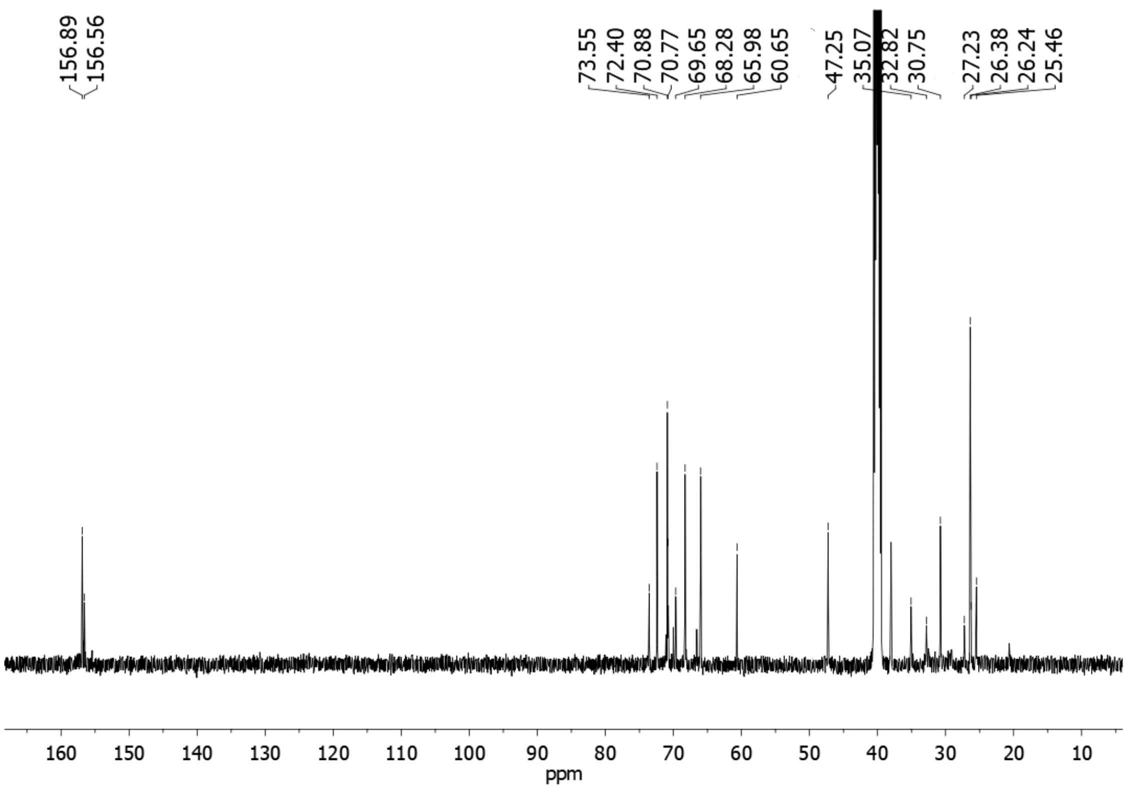

Figure S14. IR spectrum of PHU **1c**.

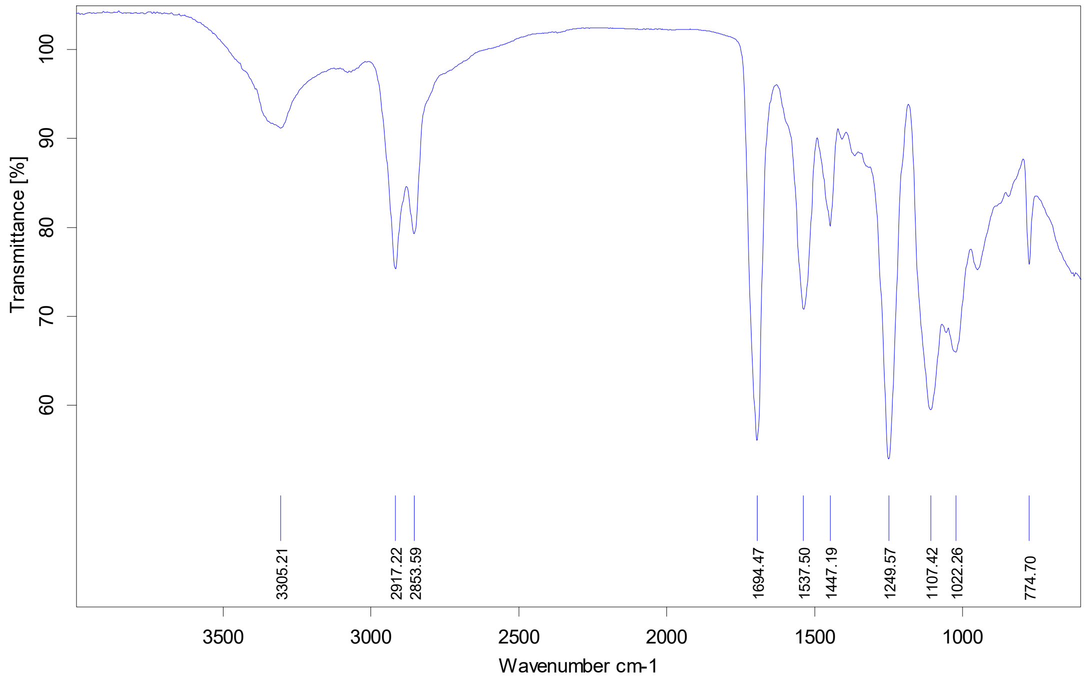

Figure S15. DSC thermogram of PHU **1c**.

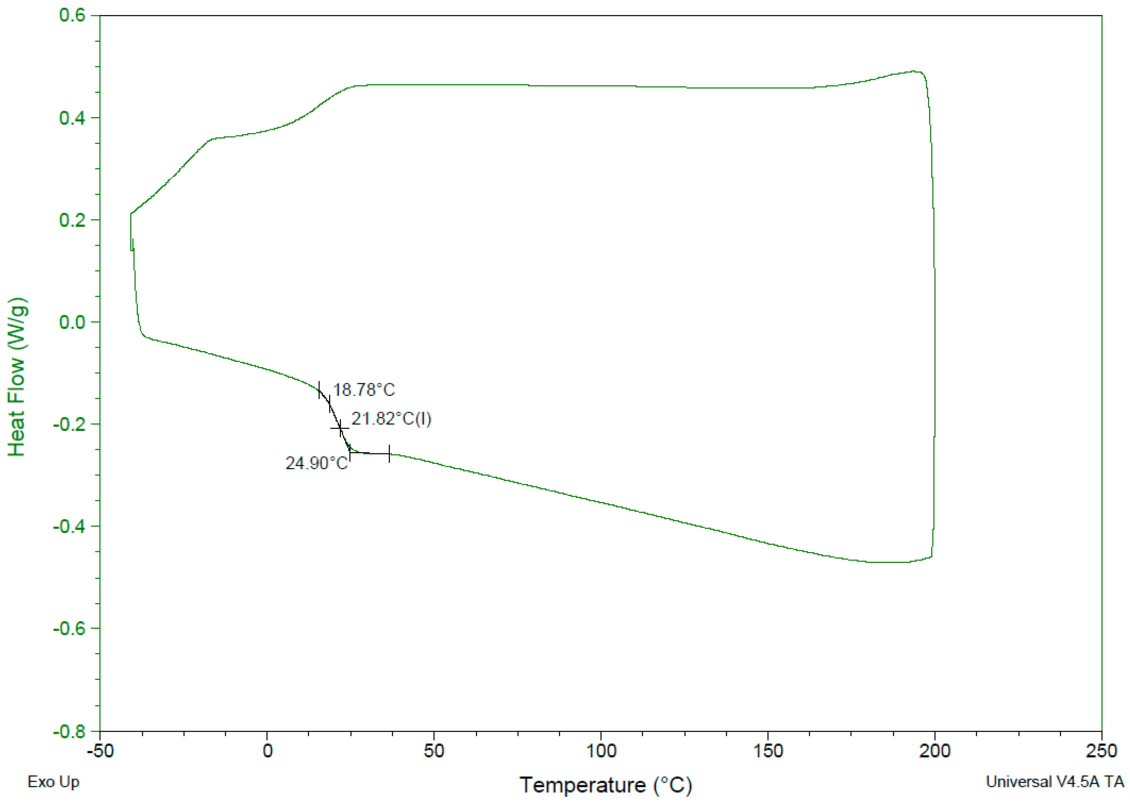

Figure S16. TGA thermogram of PHU **1c**.

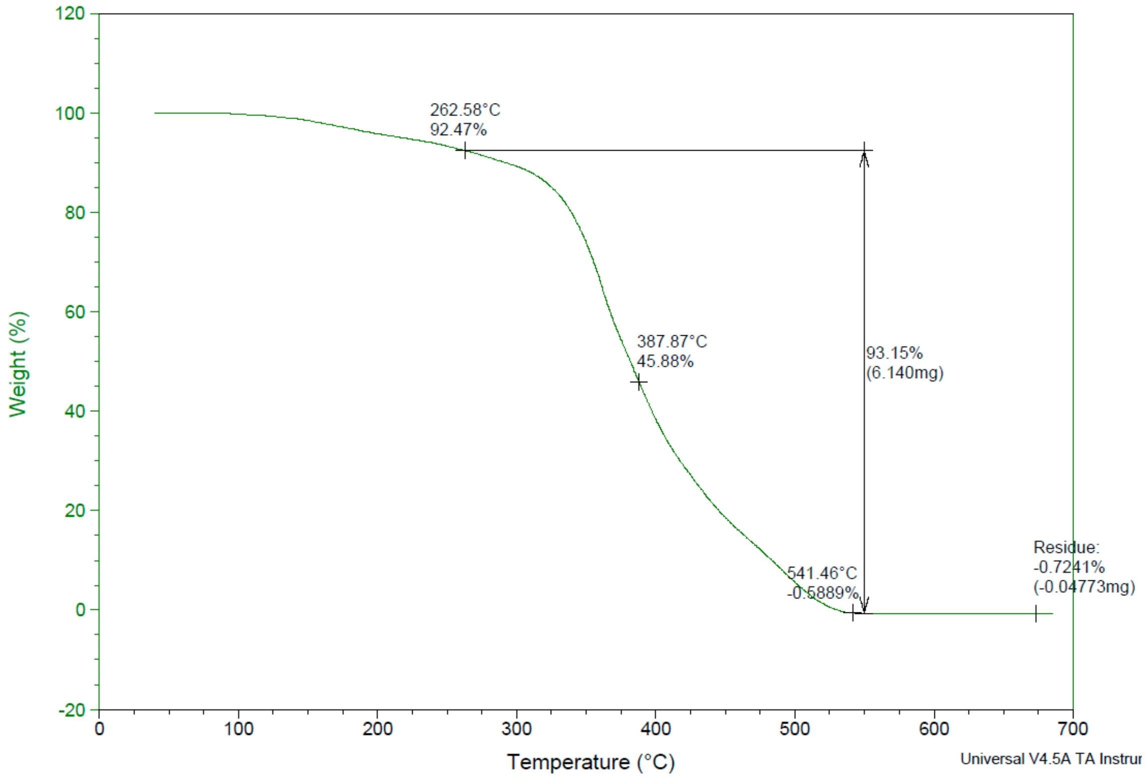

Figure S17. ^{1}H-NMR spectrum of PHU **1d** in DMSO-d6.

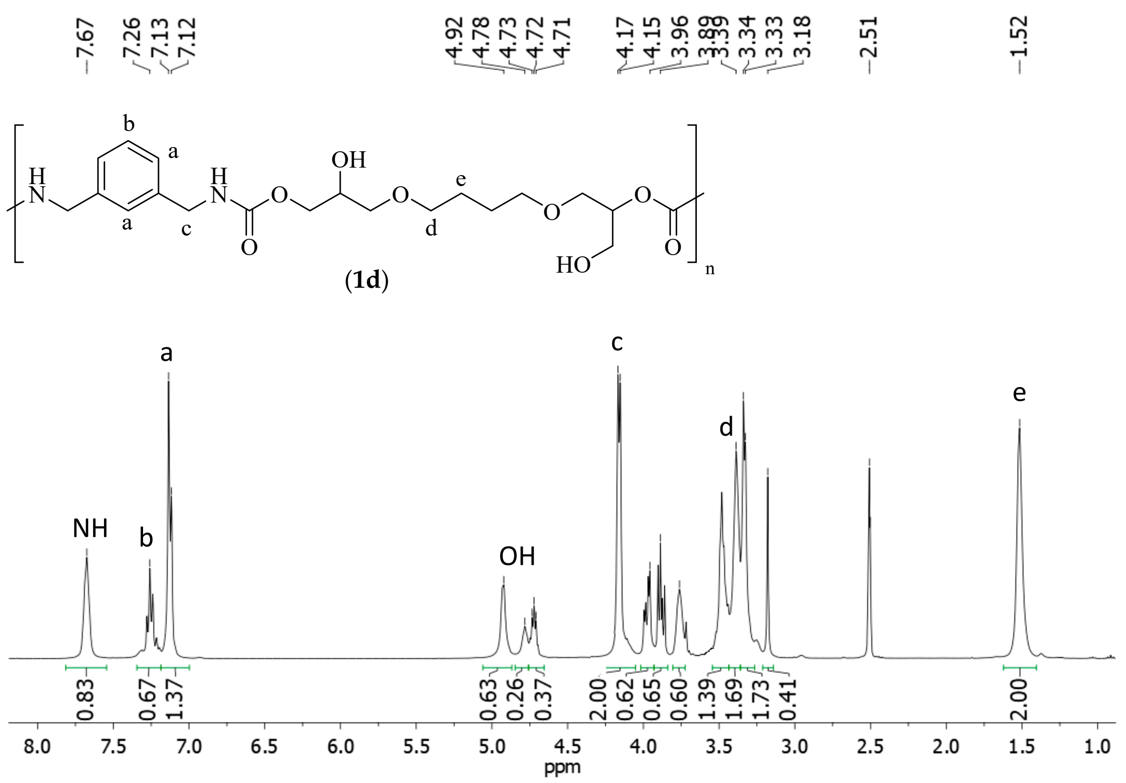

Figure S18. ^{13}C-{^{1}H}-NMR spectrum of PHU **1d** in DMSO-d6.

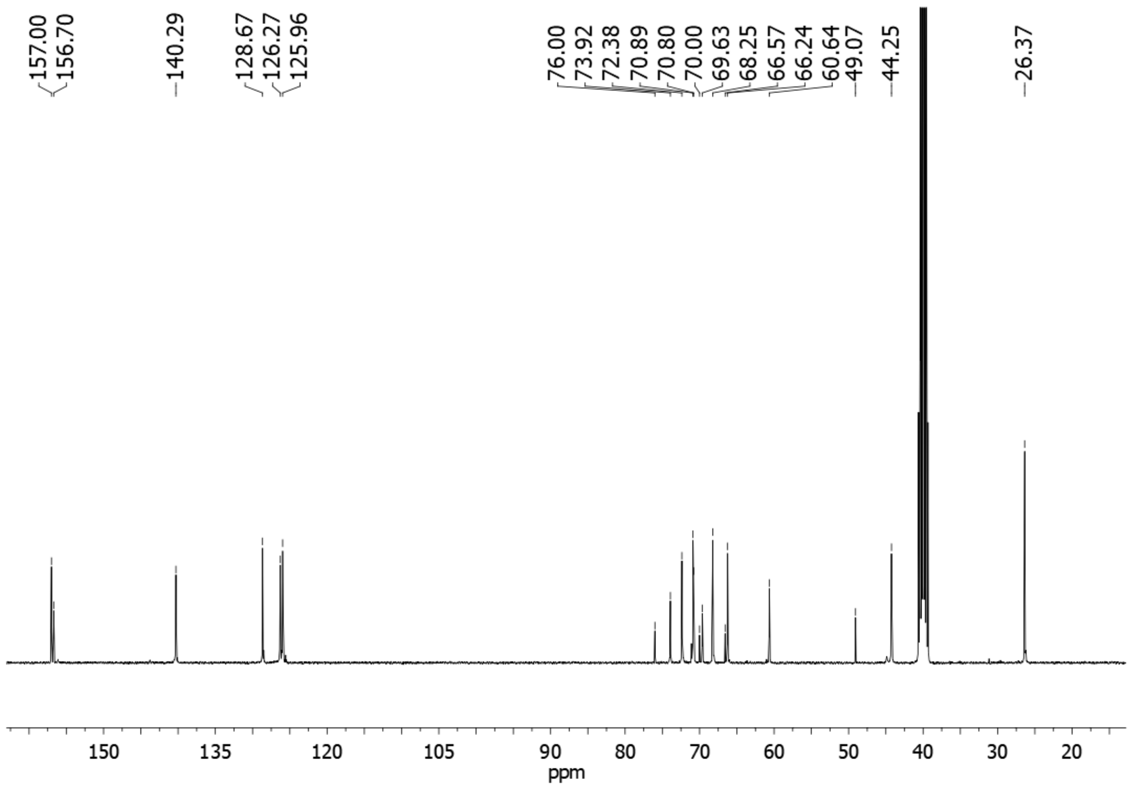

Figure S19. IR spectrum of PHU **1d**.

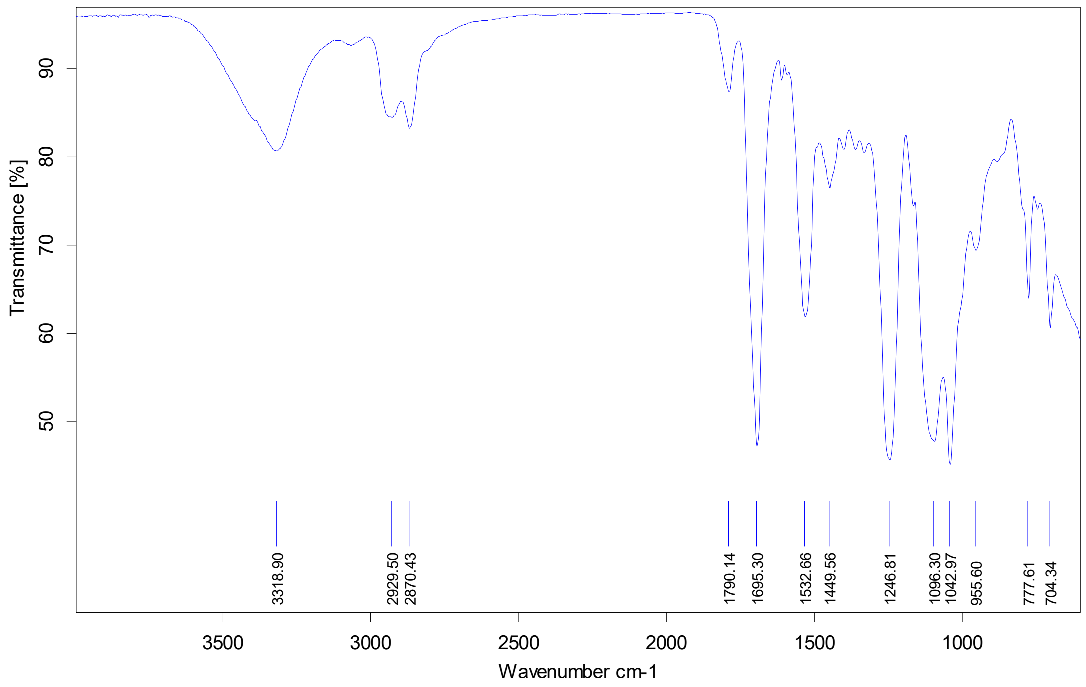

Figure S20. GPC trace of PHU **1d**.

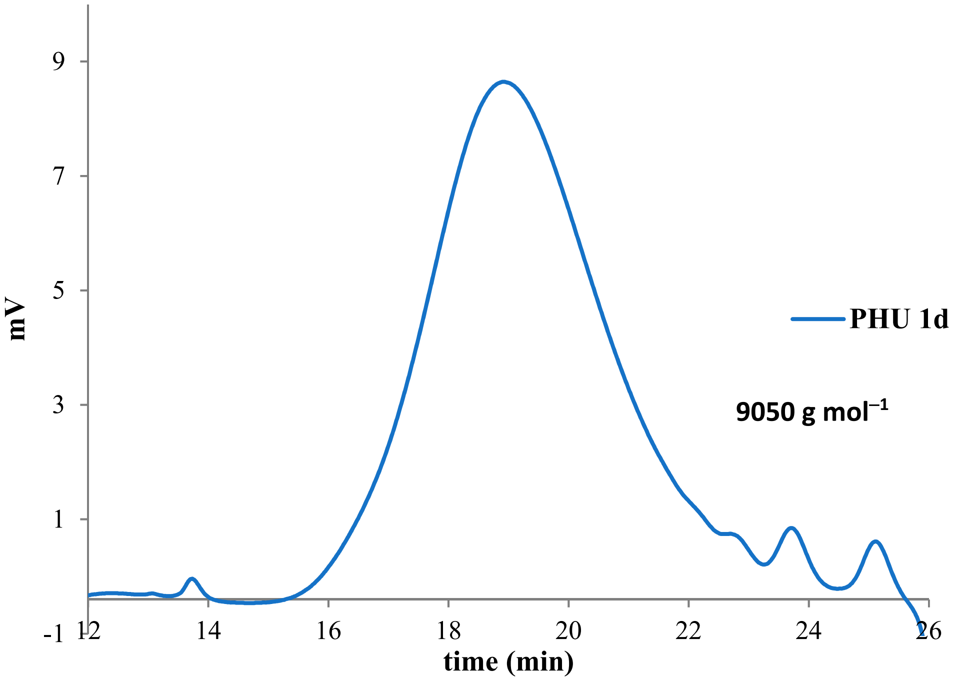

Figure S21. DSC thermogram of PHU **1d**.

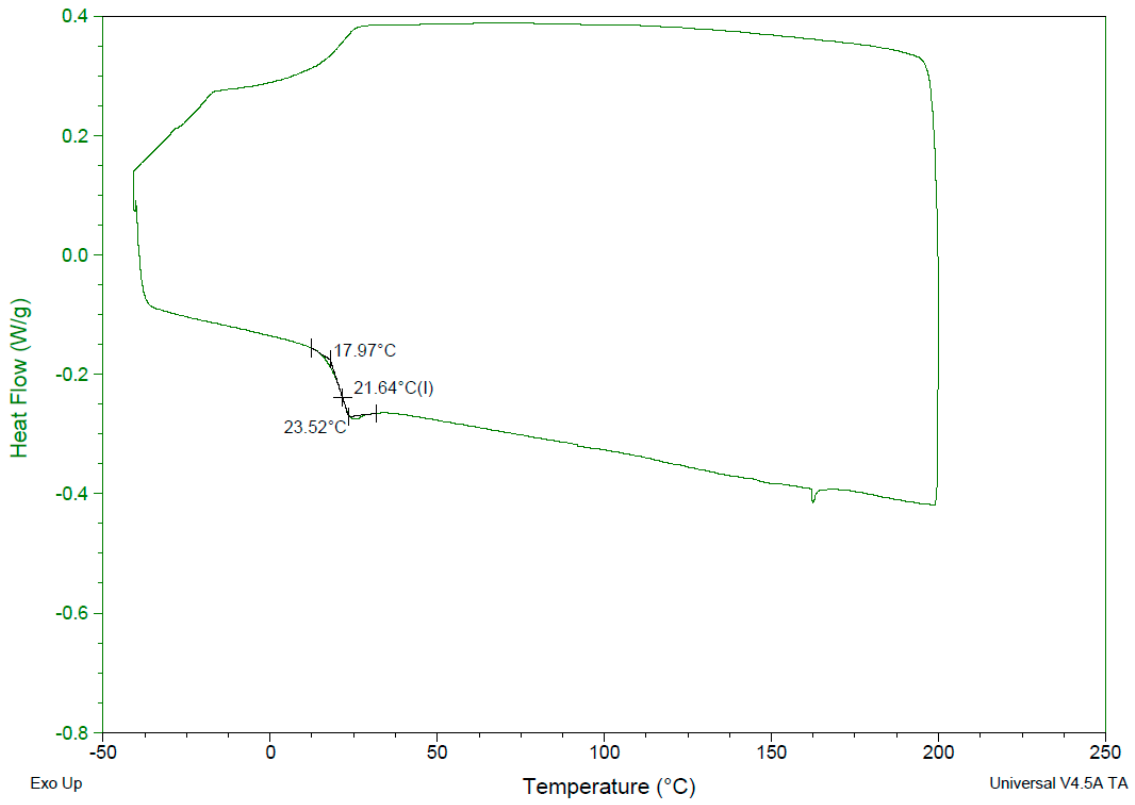

Figure S22. TGA thermogram of PHU **1d**.

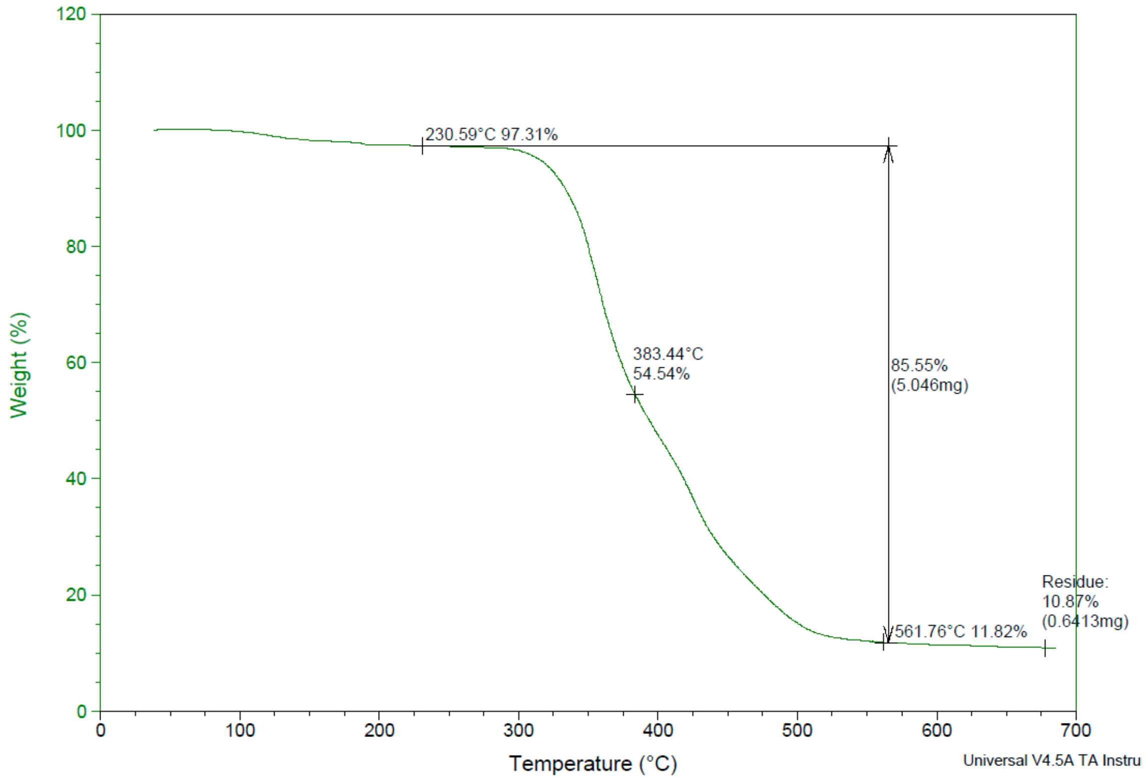

Figure S23. ^{1}H-NMR spectrum of PHU **1e** in DMSO-d_6.

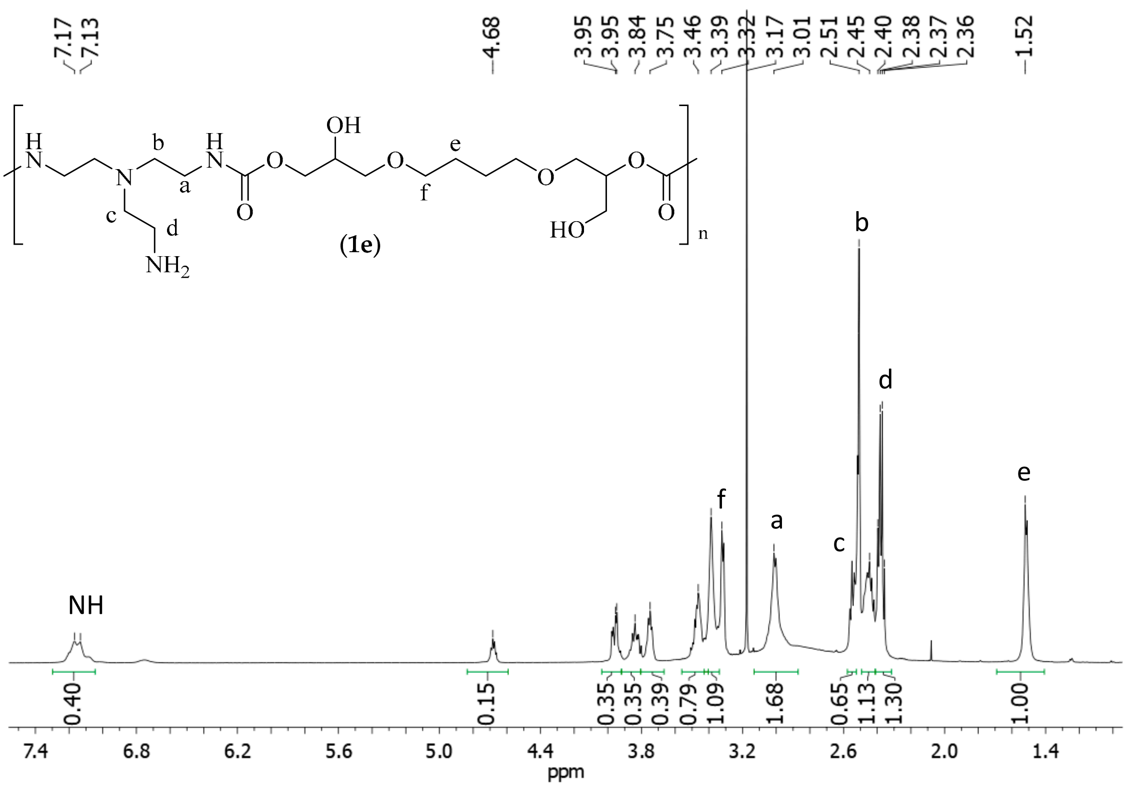

Figure S24. ^{13}C-{^{1}H}-NMR spectrum of PHU **1e** in DMSO-d_6.

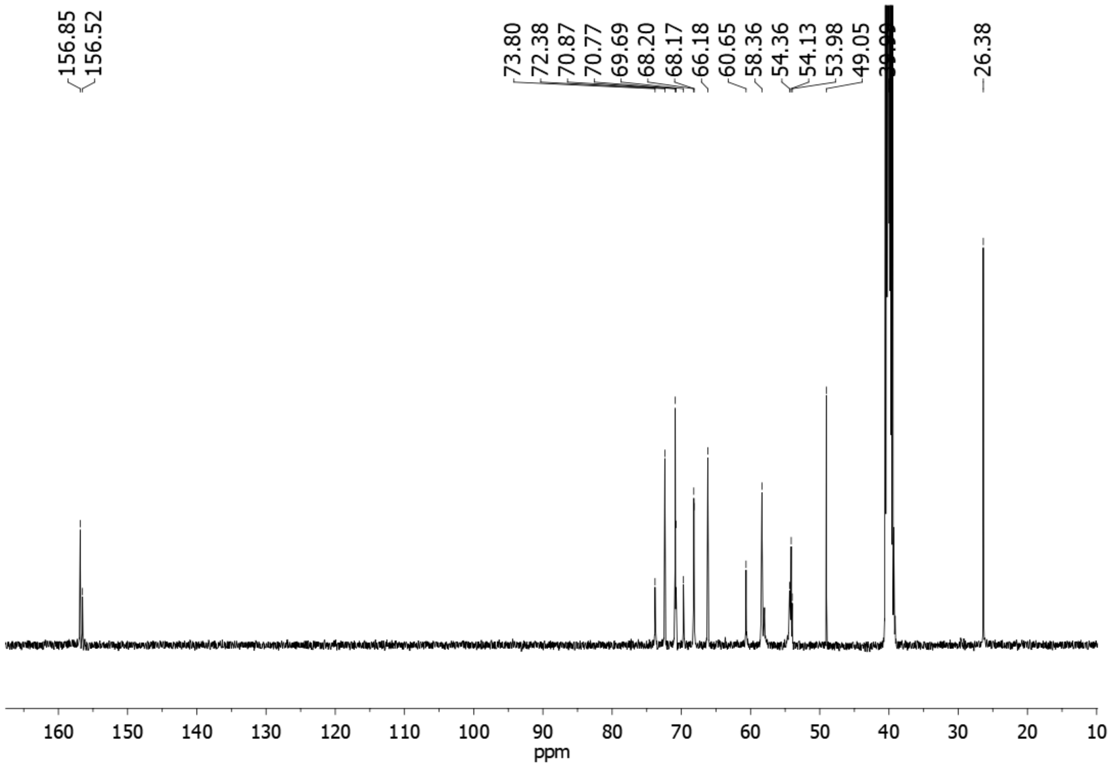

Figure S25. IR spectrum of PHU **1e**.

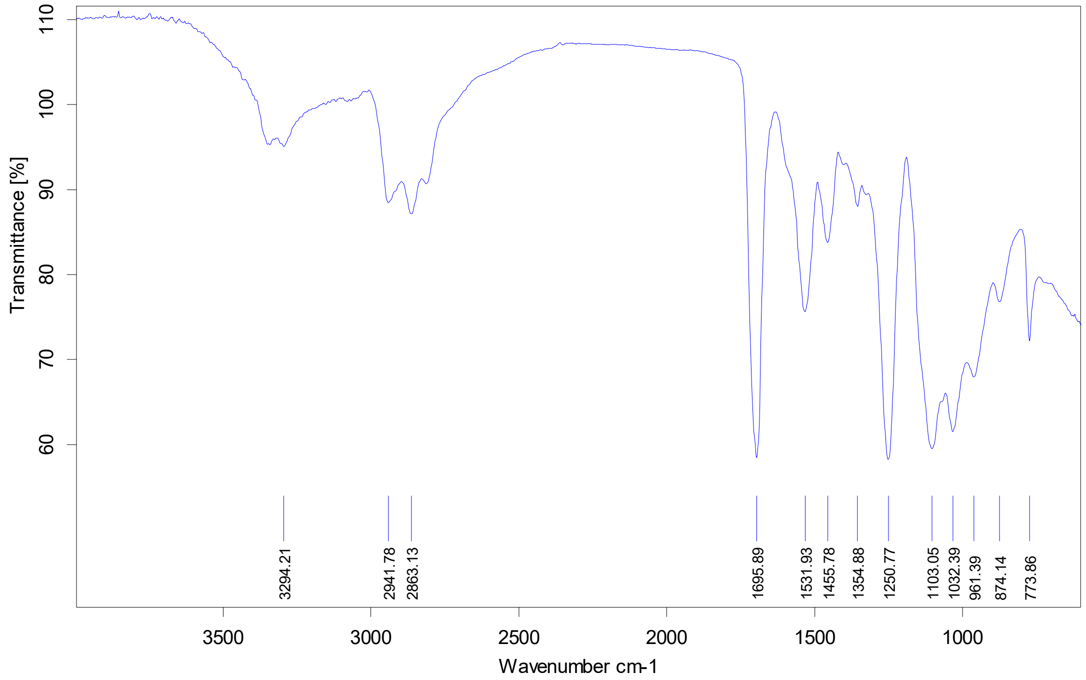

Figure S26. DSC thermogram of PHU **1e**.

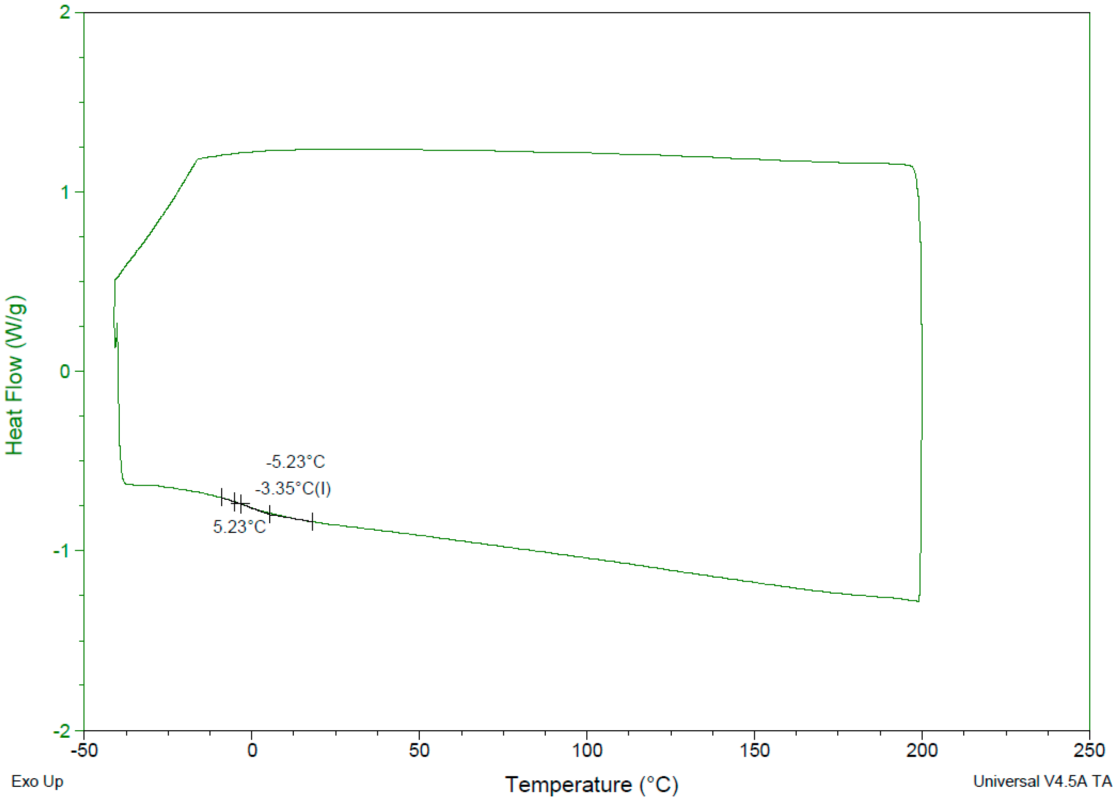

Figure S27. TGA thermogram of PHU **1e**.

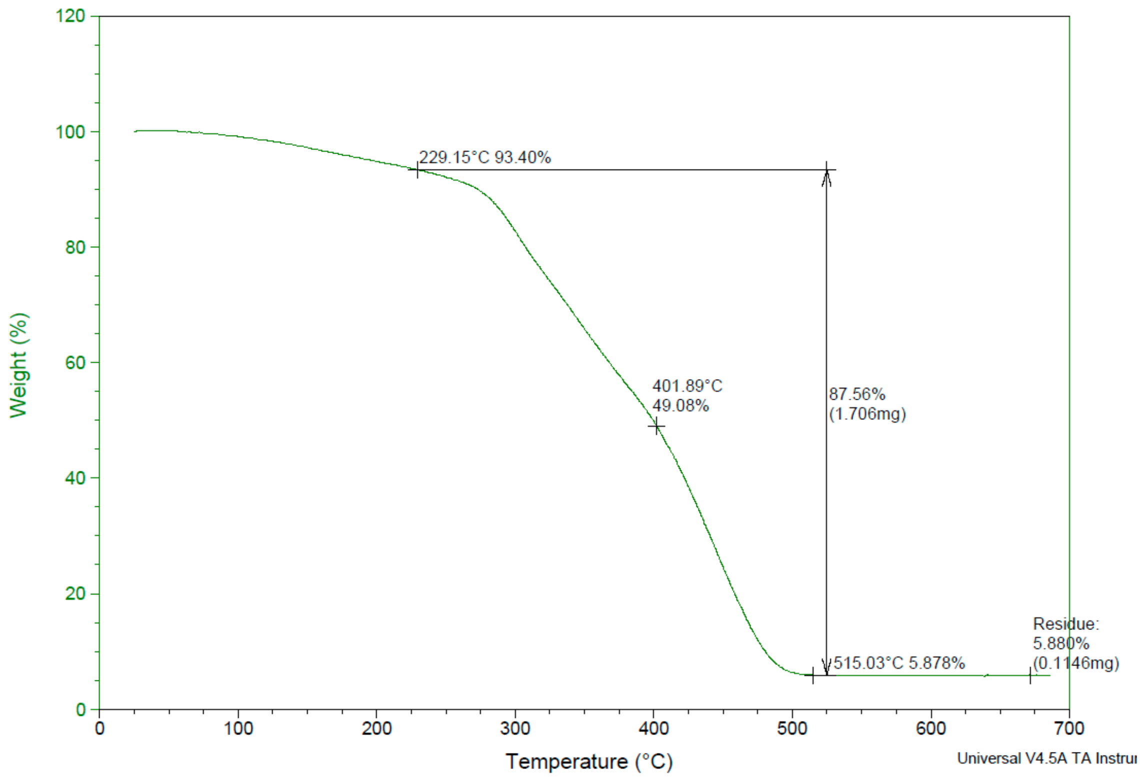

Figure S28. IR spectrum of PHU **1e** crosslinked (0.66 eq. tris-(aminoethyl)amine).

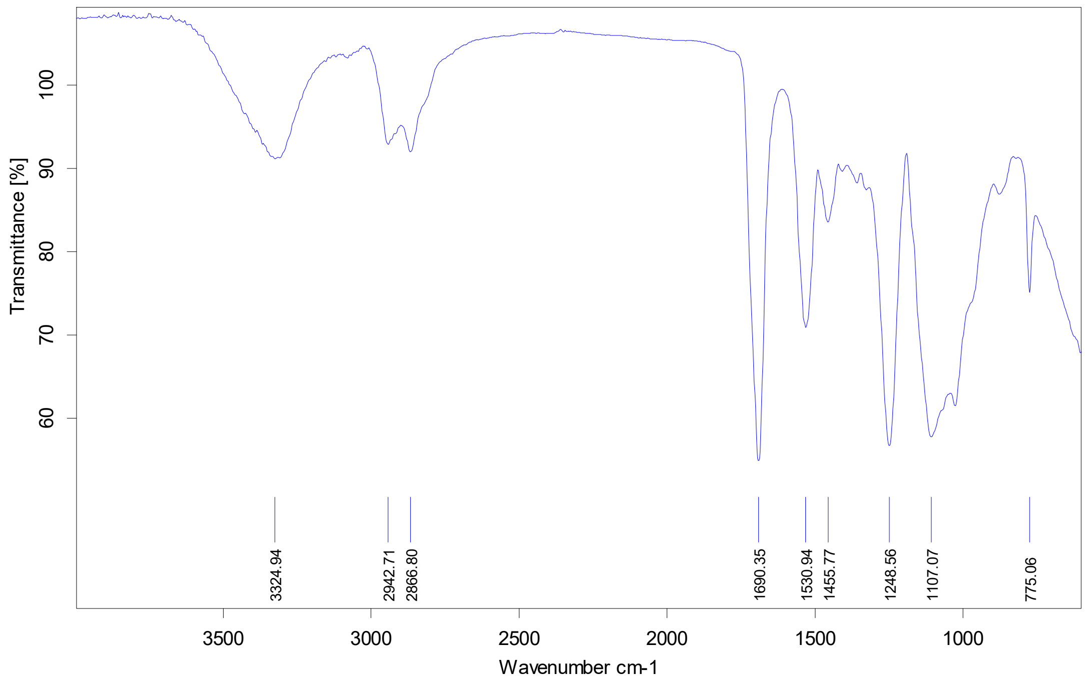

Figure S29. DSC thermogram of PHU **1e** crosslinked (0.66 eq. tris-(aminoethyl)amine).

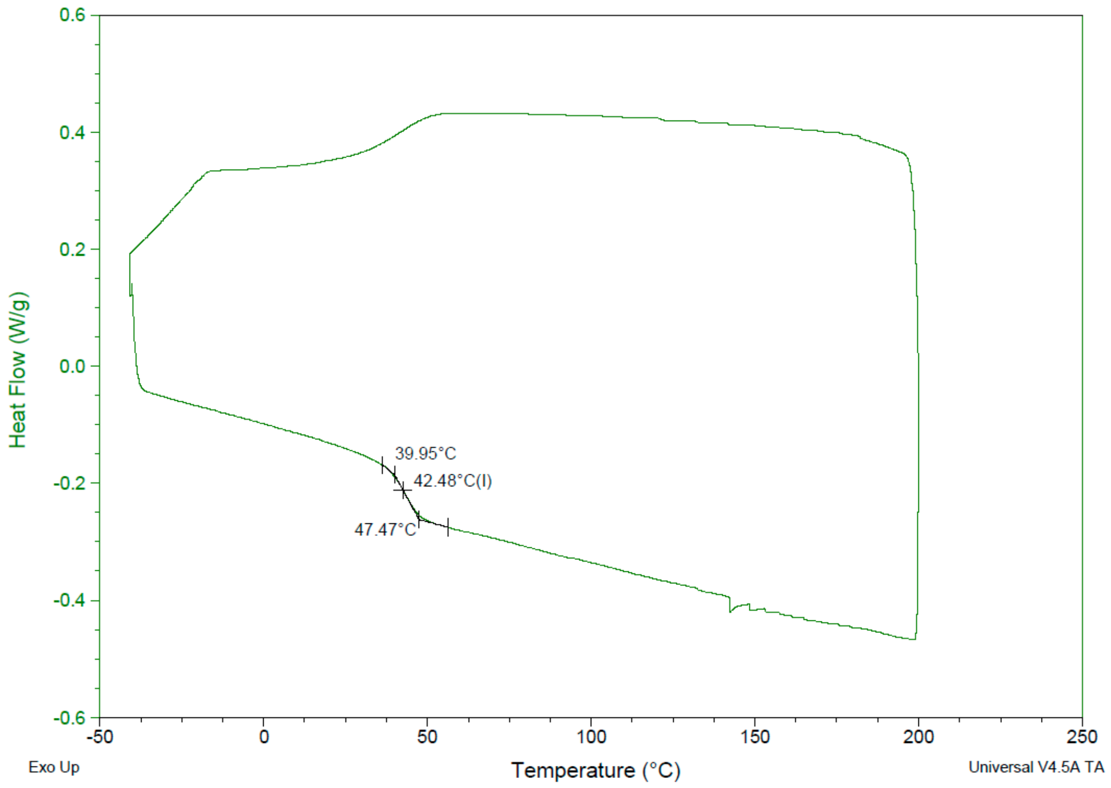

Figure S30. TGA thermogram of PHU **1e** crosslinked (0.66 eq. tris-(aminoethyl)amine).

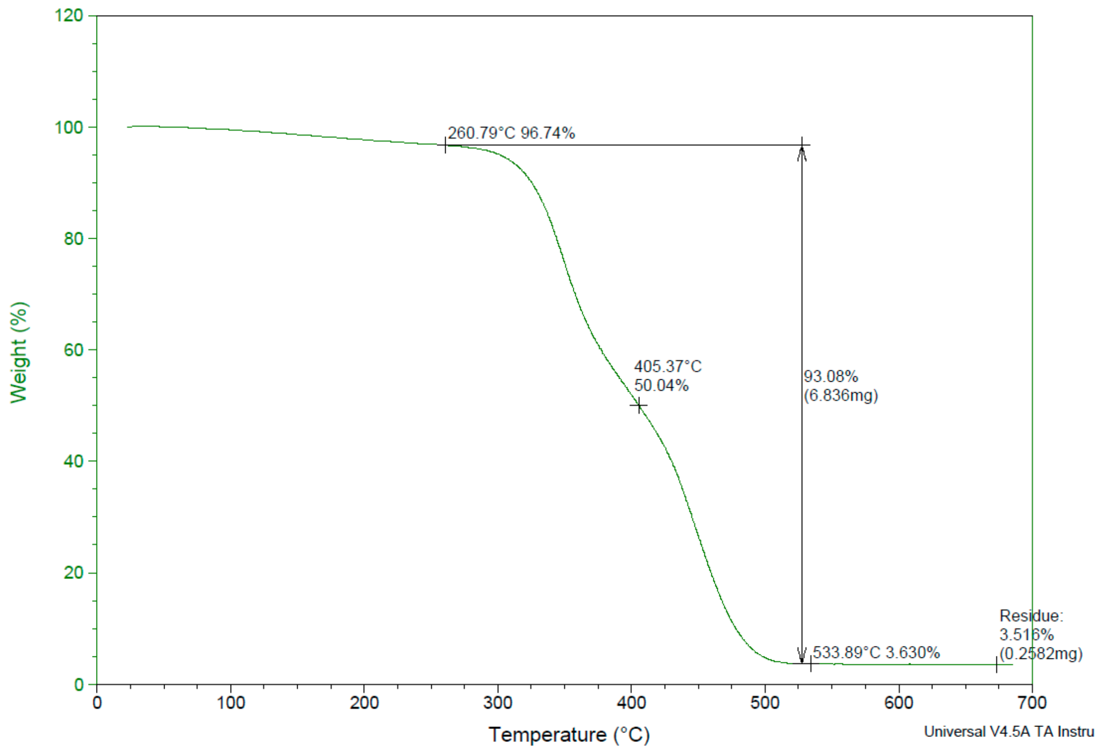

Figure S31. ^{1}H-NMR spectrum of PHU **1f** in DMSO-d$_6$.

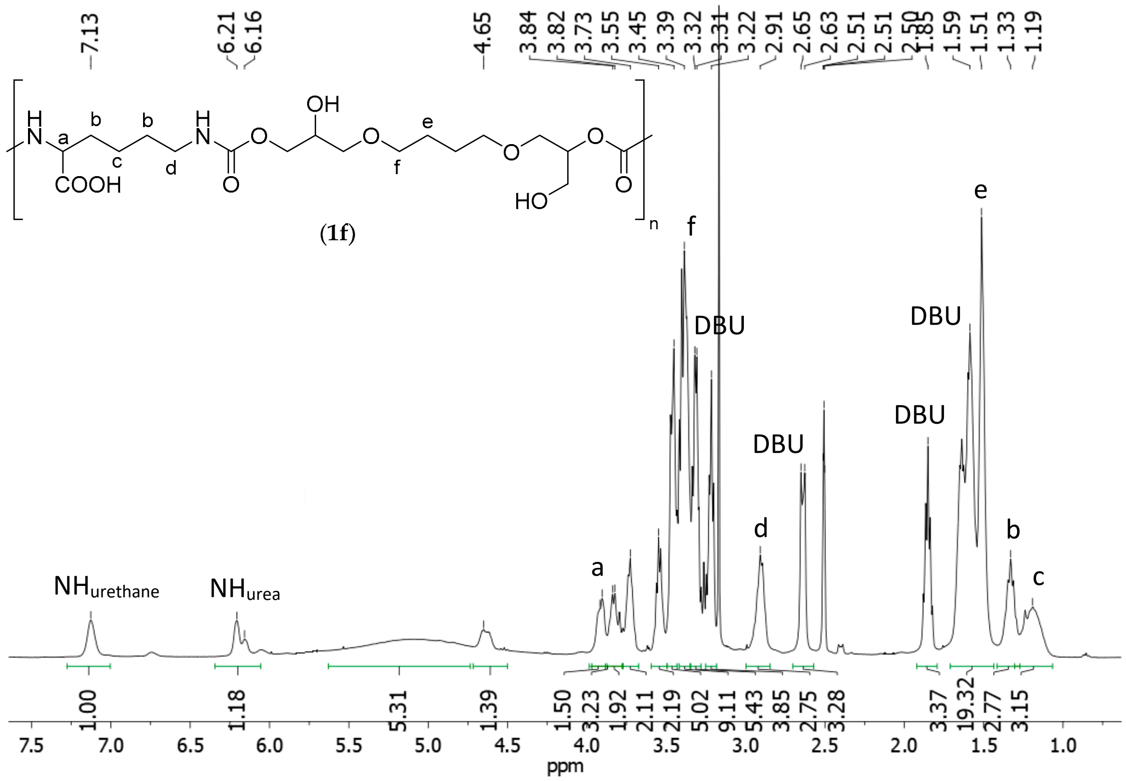

Figure S32. ^{13}C-{^{1}H}-NMR spectrum of PHU **1f** in DMSO-d$_6$.

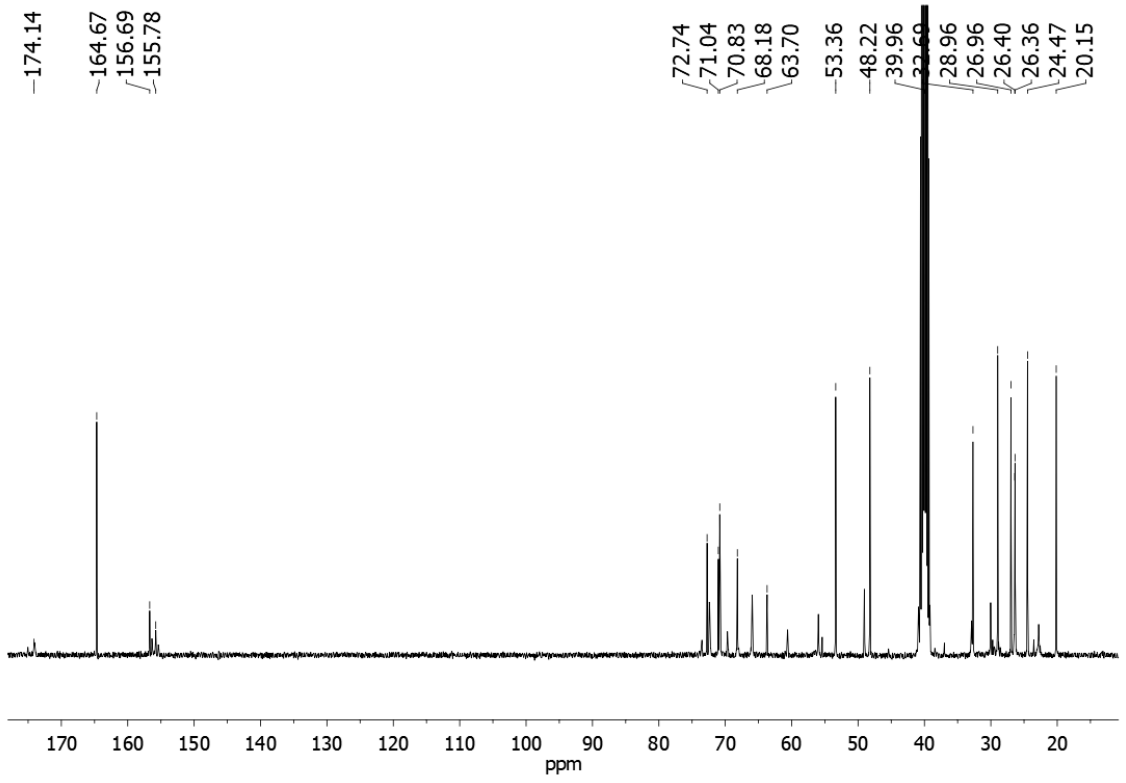

Figure S33. IR spectrum of PHU **1f**.

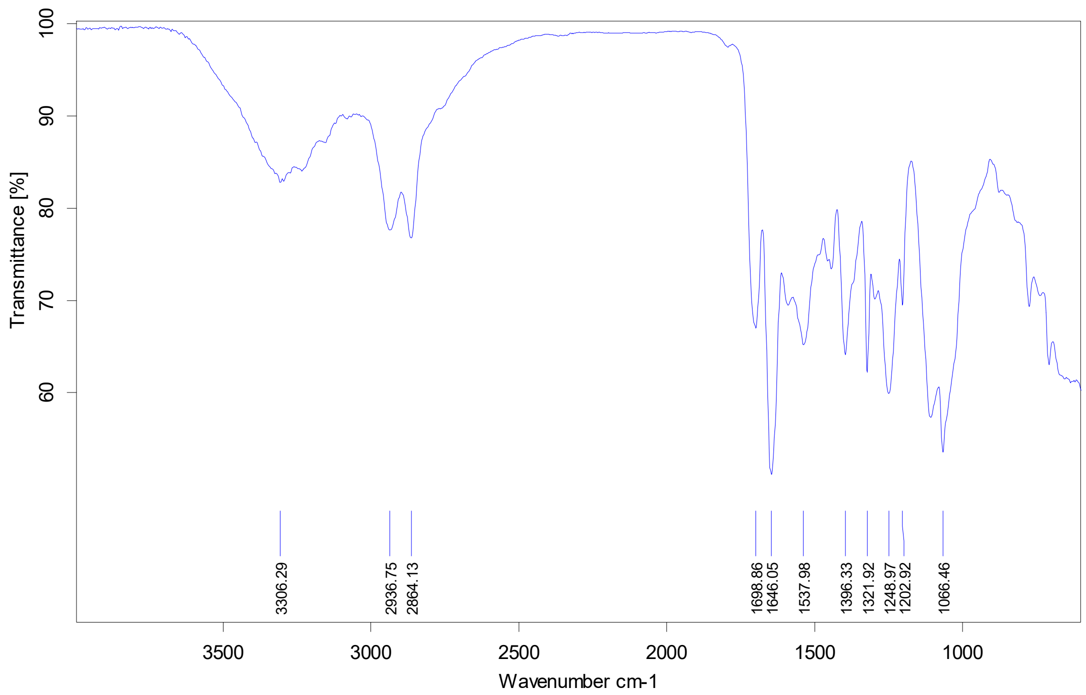

Figure S34. GPC trace of PHU **1f**.

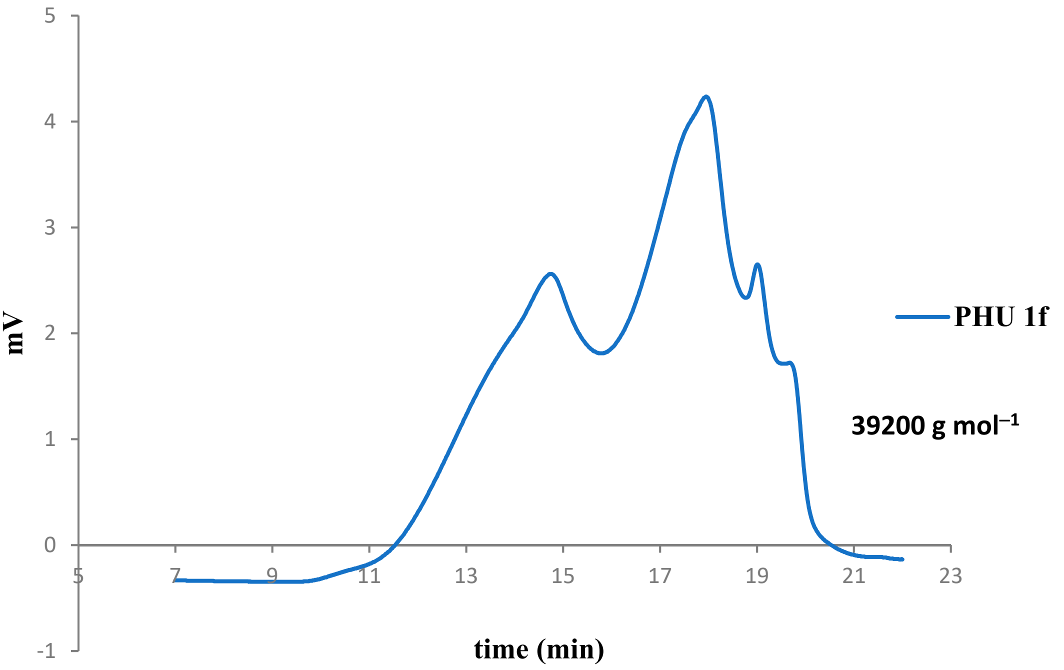

Figure S35. DOSY spectrum of PHU **1f.**

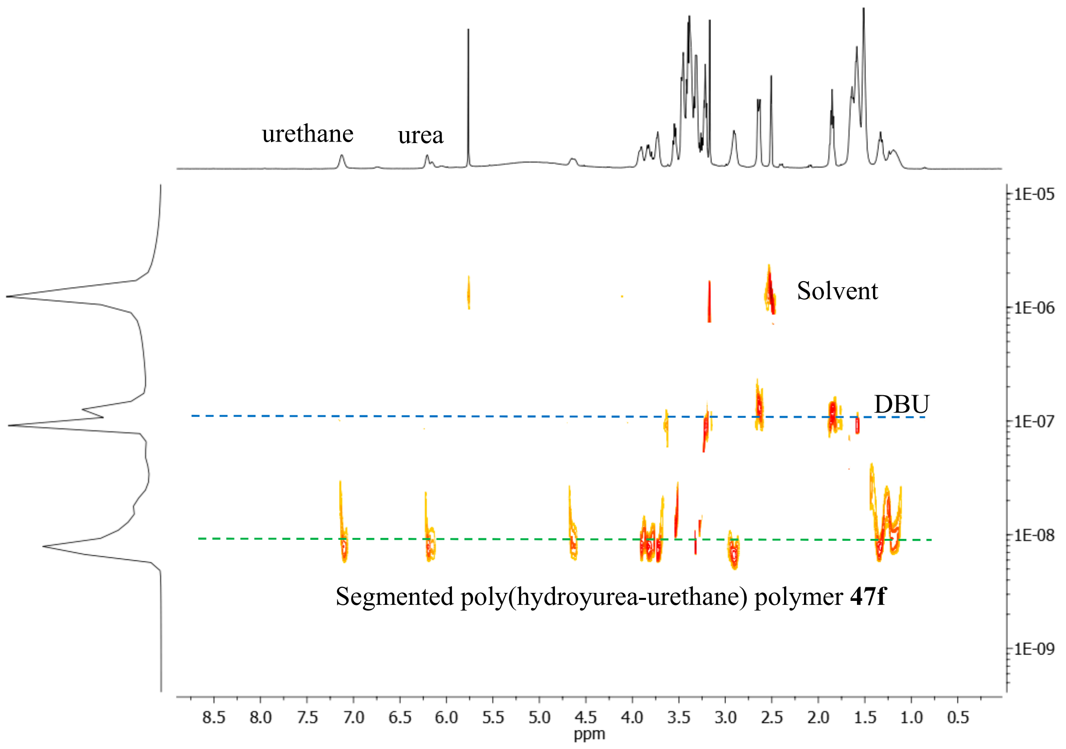

Figure S36. DSC thermogram of PHU **1f.**

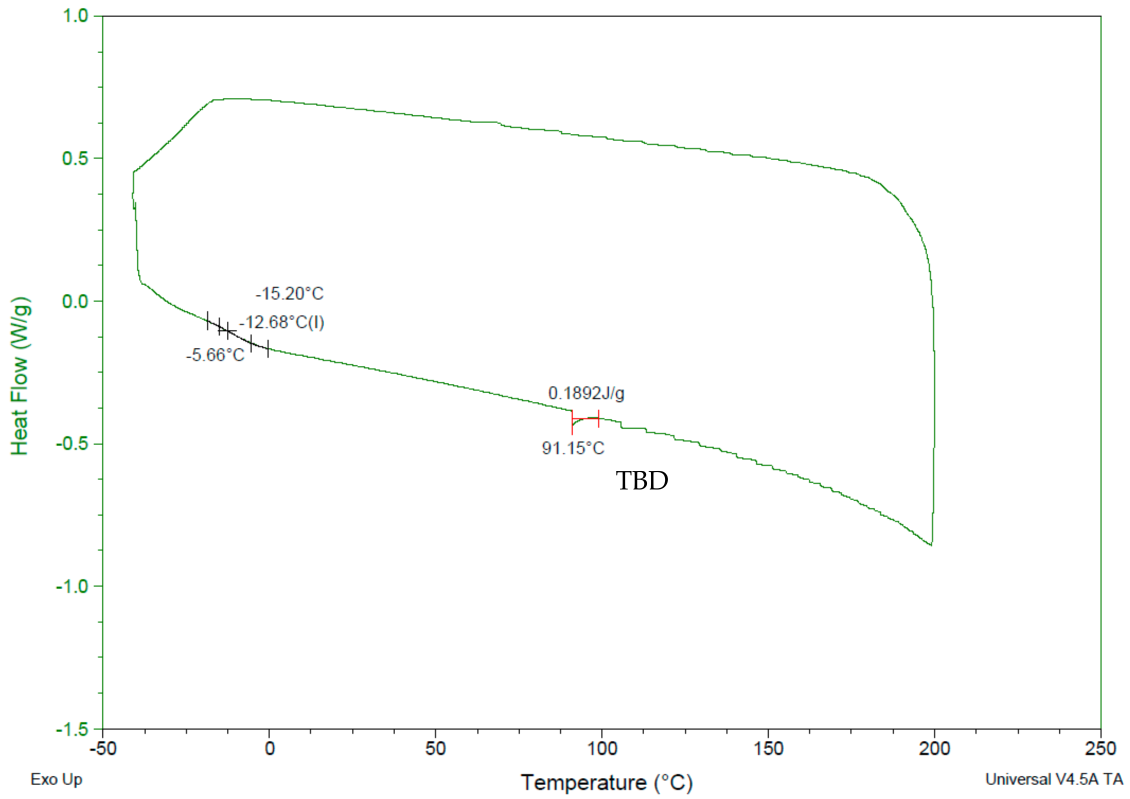

Figure S37. TGA thermogram of PHU **1f.**

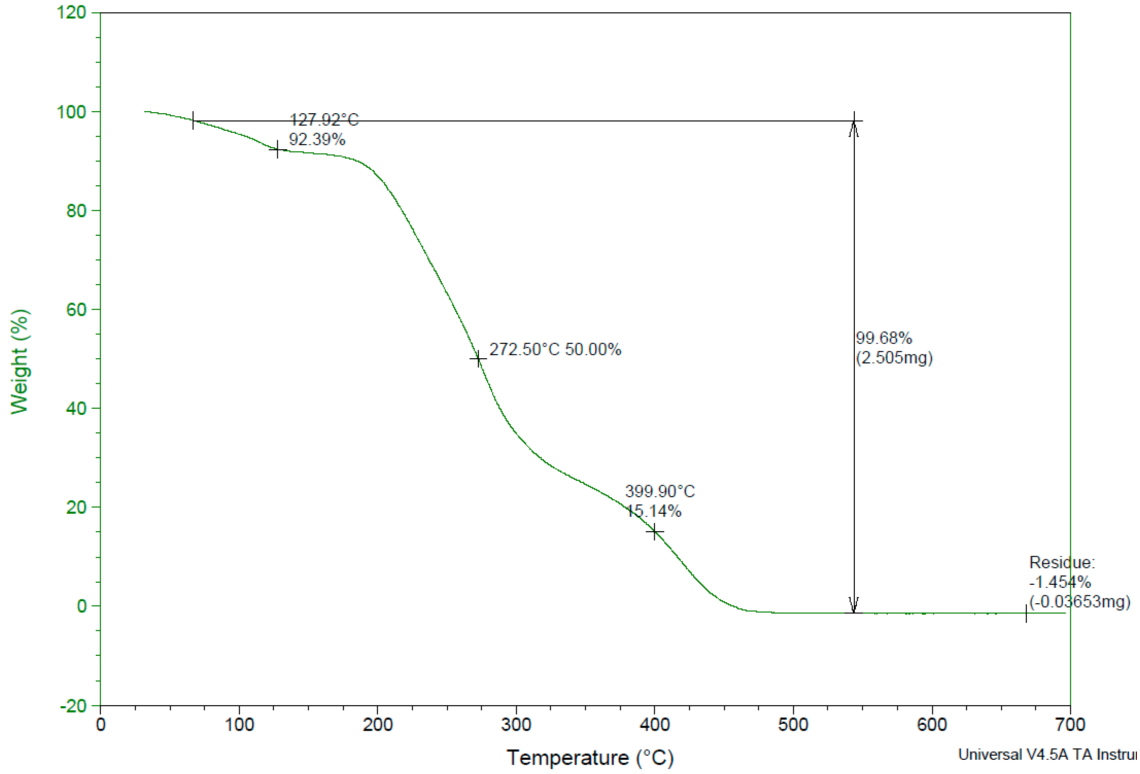

Figure S38. ^{1}H-NMR spectrum of hydroxycarbamate **2** in DMSO-d6.

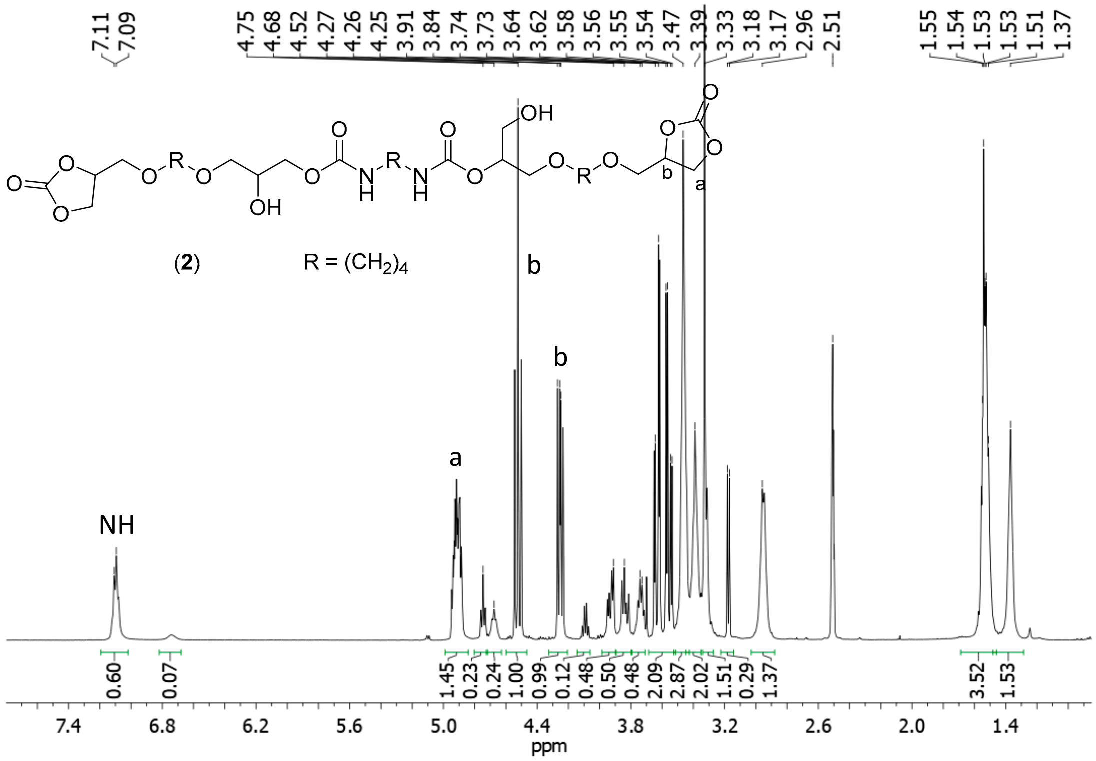

Figure S39. ^{13}C-{^{1}H}-NMR spectrum of hydroxycarbamate **2** in DMSO-d$_6$.

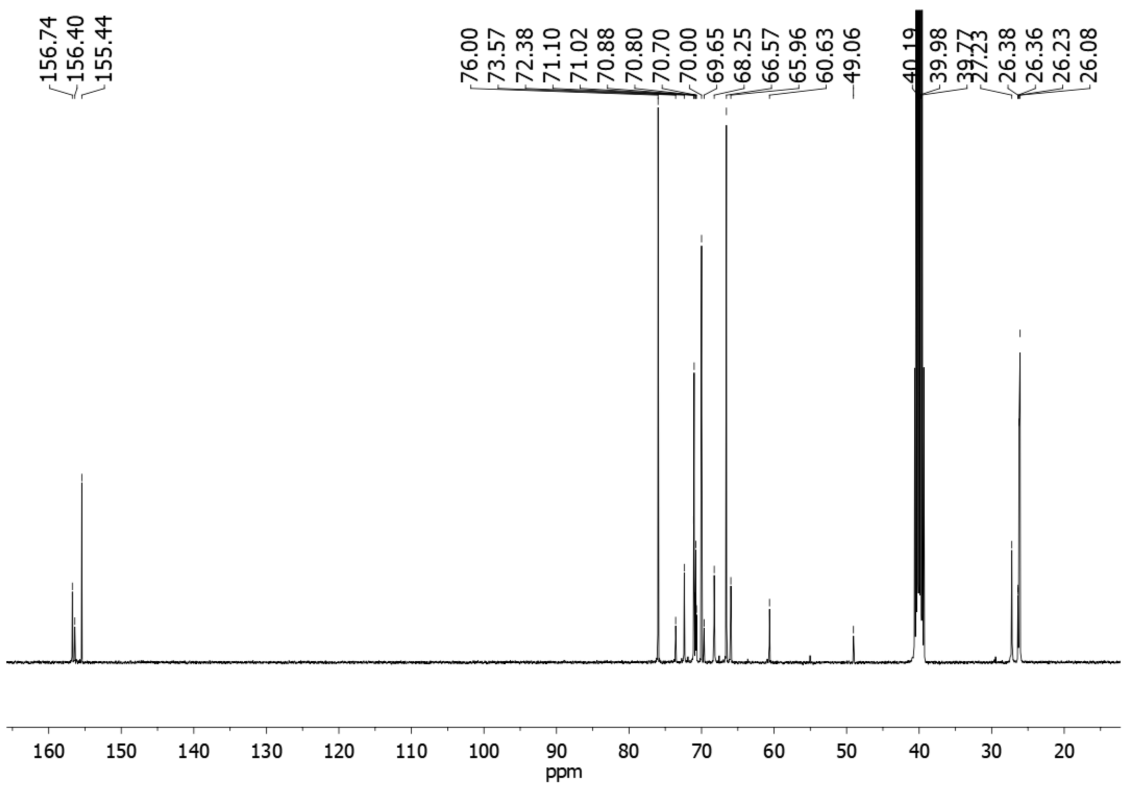

Figure S40. IR spectrum of hydroxycarbamate **2**.

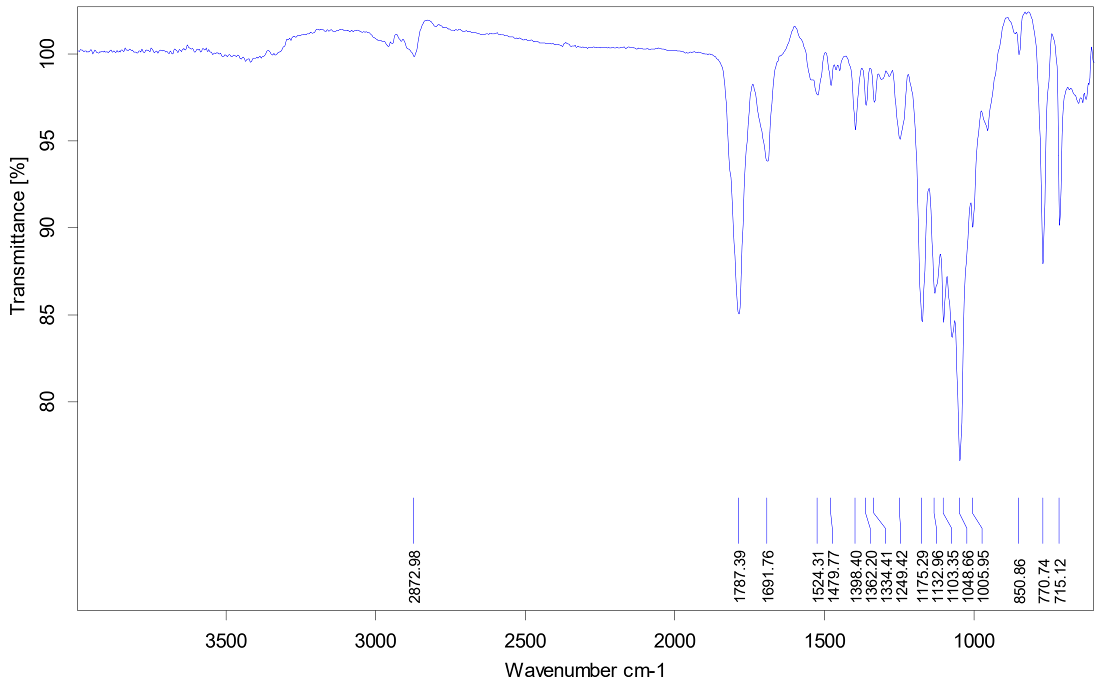

Figure S41. MALDI-ToF spectrum of hydroxycarbamate **2**.

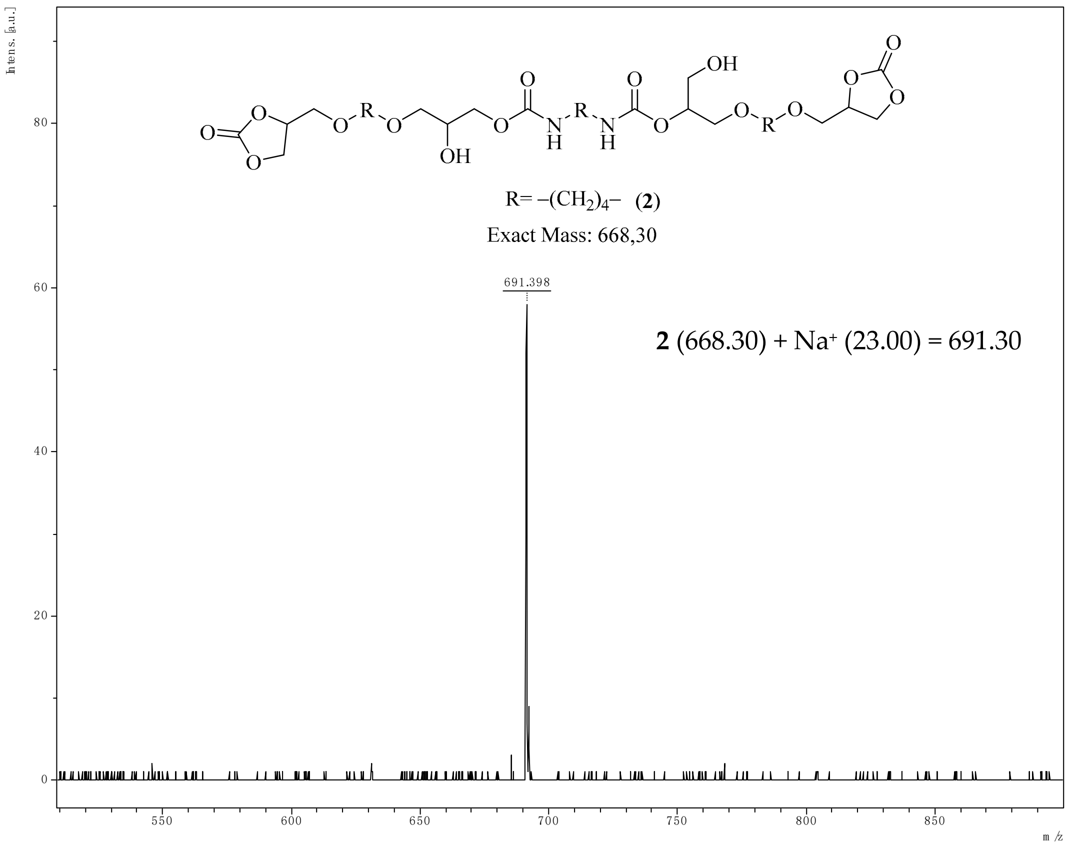

Figure S42. ^{1}H-NMR spectrum of hydroxycarbamate **3** in DMSO-d$_6$.

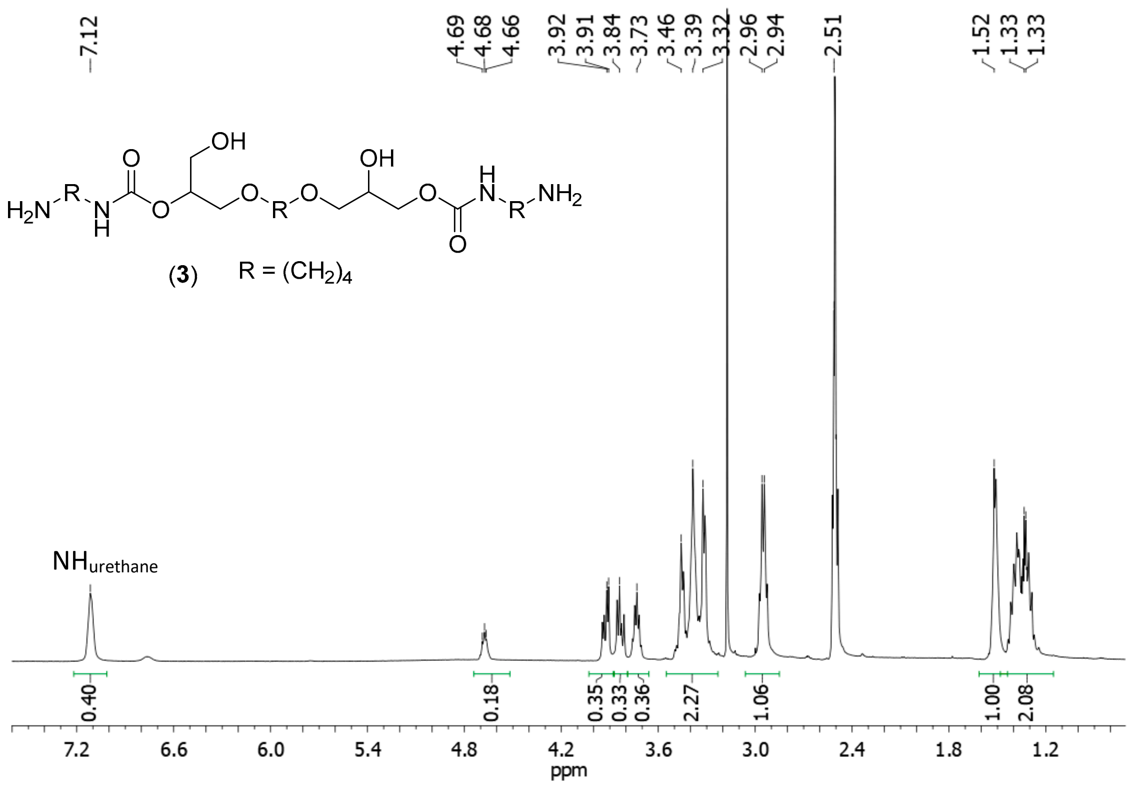

Figure S43. ^{13}C-{^{1}H}-NMR spectrum of hydroxycarbamate **3** in DMSO-d_6.

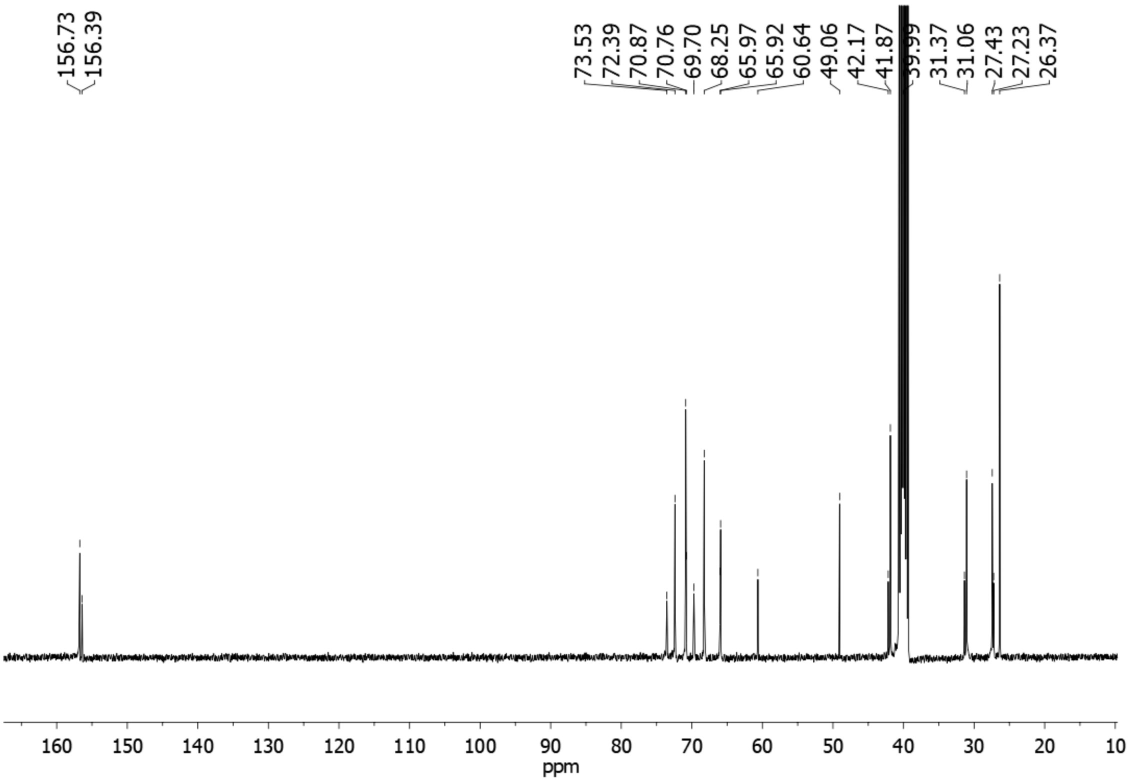

Figure S44. IR spectrum of hydroxycarbamate **3**.

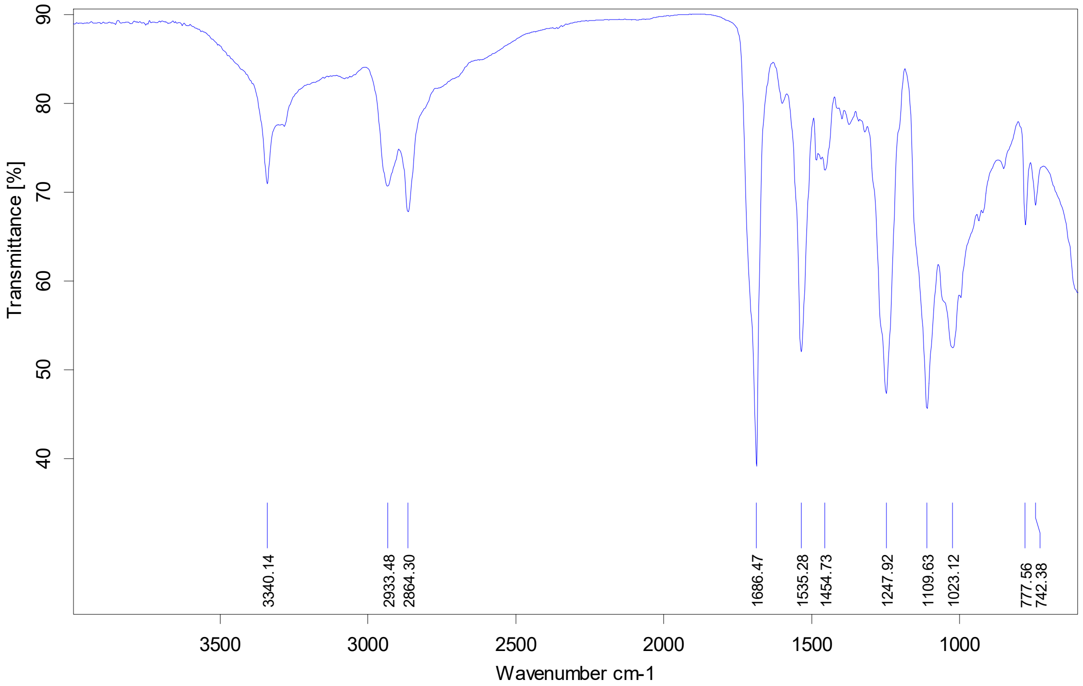

Figure S45. MALDI-ToF spectrum of hydroxycarbamate **3**.

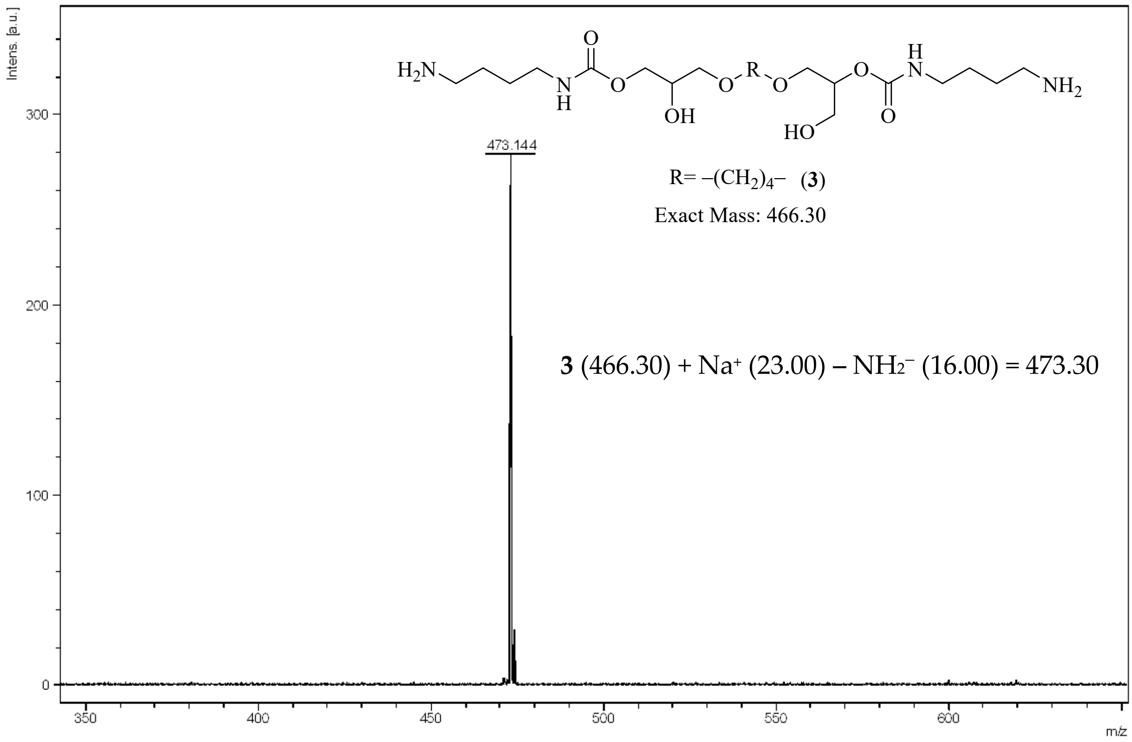

Supplement: Supplementary file 1 [file polymers-14-02719-s001.zip › polymers-1779277-supplementary.pdf]
